# Engagement or disengagement: understanding the emotional dynamics of EFL learners in GenAI-assisted writing from the sociocultural perspective

**DOI:** 10.3389/fpsyg.2026.1934782

**Published:** 2026-10-21

**Authors:** Keqing Wu, Miao Yang, Changmin Lin

**Affiliations:** 1 Department of Foreign Languages, Shantou University Medical College, Shantou, China; 2 Office of Academic Affairs, Shantou University Medical College, Shantou, China

**Keywords:** EFL writing, emotional engagement, Generative AI, mediation, perezhivanie, sociocultural theory

## Abstract

**Introduction:**

Generative artificial intelligence (GenAI) has reshaped EFL writing education, yet the emotional dimensions of student engagement in GenAI-assisted writing remain underexplored. This longitudinal multiple-case study investigates how Chinese EFL learners' emotional engagement evolves during a GenAI-assisted college writing course and what factors drive this evolution.

**Methods:**

Drawing on the sociocultural framework of L2 student engagement with written feedback, we traced the emotional trajectories of three focal students across three writing tasks (exposition, group feature article, and argumentation) within a 14-week course. Data were collected through semi-structured interviews with stimulated recall, reflective reports, GenAI-student chatlogs, drafts, informal chats, and course syllabus, and analyzed via thematic analysis.

**Results:**

Our findings reveal three qualitatively distinct trajectories: positive-resilient, characterized by sustained positive affect with temporary fluctuations; pragmatic-transformative, marked by a gradual shift from practical disengagement to critical engagement; and vulnerable-contingent, moving from moderate disengagement, through tentative peer-mediated improvement, to eventual breakdown and intensified disengagement. These trajectories were shaped by the dialectical interplay between individual factors (e.g., English proficiency, GenAI literacy, mindsets, beliefs, and emotion regulation) and environmental factors (GenAI-relevant task design, peer and teacher mediation, and institutional policies).

**Discussion:**

The study extends the sociocultural framework by identifying English proficiency and GenAI literacy as critical developmental competencies, embedding GenAI-related factors across multiple environmental levels, and foregrounding emotional engagement as a dynamic, fluid state that interacts with behavioral and cognitive engagement over time. The findings challenge universalist claims about GenAI's uniform affective benefits and underscore the need for differentiated pedagogical approaches that attend to learners' diverse emotional needs in GenAI-mediated writing contexts.

## 1 Introduction

Generative artificial intelligence (GenAI)^1^ has rapidly transformed English as a Foreign Language (EFL) writing education, with recognized cognitive and technical benefits (Chan and Hu, 2023; Kohnke and Moorhouse, 2025). Unlike traditional automated writing evaluation systems that primarily addressed surface-level errors, GenAI platforms such as ChatGPT provide conversational, generative, and context-sensitive support across the entire writing process—from pre-writing ideation and drafting to revision and final editing (Barrot, 2023; Yan and Zhang, 2024). Recent research has identified foundational competencies—critical AI literacy and self-efficacy—that learners must marshal to navigate this reconfigured writing landscape (Jin et al., 2025; Yao and Fan, 2026a,b). However, while the cognitive affordances of these tools have been extensively documented, the emotional dimensions of student engagement with GenAI-assisted writing remain comparatively underexplored.

Emotional engagement, which encompasses learners' affective and attitudinal responses to writing tasks, tools, and instructional contexts, constitutes a critical component of L2 writing development (Ma et al., 2026; Yeung, 2025). Empirical studies have established that GenAI-assisted writing can both positively and negatively influence learners' emotional experiences. On the positive side, it has been shown to reduce student anxiety (Hawanti and Zubaydulloevna, 2023; Mahapatra, 2024; Song and Song, 2023), increase confidence (Su et al., 2023; Wang and Dang, 2024), and enhance motivation and wellbeing (Kohnke and Moorhouse, 2025). On the negative side, the black-box nature of GenAI suggestions, potential for factual inaccuracies and ease of automated text generation may provoke anxiety about academic integrity, de-skilling, or alienation from the writing process (Yan, 2025; Yao and Liu, 2026). The emotional labor of prompt engineering can engender either frustration or a sense of accomplishment (Li et al., 2026; Yan and Zhang, 2024), and some students may miss the empathy and nuanced understanding that human teachers provide (Geng and Yu, 2024; Yao and Liu, 2026). These divergent findings suggest that affective responses are not uniform but contingent on individual and contextual conditions (Pekrun and Linnenbrink-Garcia, 2012; Saeli and Rahmati, 2023; Xu and Zhang, 2025).

Despite this growing body of evidence, significant gaps remain. First, existing research has focused predominantly on students' emotional reactions to post-writing GenAI feedback, largely overlooking the formative affective experiences that emerge during the pre-writing and drafting stages where GenAI tools are increasingly active. Second, longitudinal studies tracking emotional dynamics across the writing process remain scarce, leaving the question of how emotional engagement evolves over time largely unanswered. Third, the individual and contextual factors shaping emotional engagement have typically been examined in a fragmented manner (Mao and Lee, 2022), treating learner variables as isolated, static entities rather than as an interconnected system. Therefore, traditional engagement models may need to be reconceptualized or extended to capture the unique interactional and affective dynamics inherent in GenAI-assisted writing (Qian and Li, 2026).

To address these gaps, the present study draws on Mao and Lee's (2022) sociocultural framework, which conceptualizes engagement as a socially situated and evolving phenomenon. Sociocultural theory (SCT) offers a critical lens for capturing the longitudinal and context-sensitive nature of emotional engagement, emphasizing the dialectical interplay between individual and environmental factors (Mao and Lee, 2022; Qian and Li, 2026). Employing a multiple-case design, we investigate how Chinese EFL learners' emotional engagement unfold during a GenAI-assisted college writing course and explore the factors that drive its evolution. By foregrounding emotional engagement as a dynamic, process-oriented construct and extending the sociocultural framework to account for GenAI-specific interactions, this research aims to provide a more holistic and dynamic understanding of the affective dimensions of GenAI-assisted writing and to inform pedagogical strategies that cultivate emotionally sustainable learning environments.

## 2 Literature review

### 2.1 Task reconfiguration and foundational competencies in GenAI-assisted writing

The recent advancement of GenAI technology has reshaped the landscape of writing education. More specifically, GenAI reconfigures EFL writing experiences through comprehensive process support (Wang and Panahi, 2023), including generating ideas, crafting outlines, composing essays, offering personalized feedback, and polishing language to enhance textual accuracy and complexity (Fuchs, 2023; Rudolph et al., 2023; Wang, 2024). This integration fundamentally alters the cognitive ecosystem of writing: the writer's role evolves from that of a sole author to an orchestrator of a complex human-AI interaction (Liu et al., 2024; Zhao, 2022), introducing entirely new cognitive activities including iterative prompt refinement, critical evaluation of AI output, strategic adapting of AI content while maintaining authorial voice (Nguyen et al., 2024; Liu et al., 2024).

Recent psychometric advancements have systematically mapped the psychological competencies required to navigate this reconfigured writing landscape. A growing body of empirical research has identified foundational constructs that underpin effective AI-assisted L2 writing. At the cognitive level, studies have decomposed the writing process into distinct cognitive activities including iterative prompt refinement, critical evaluation of AI-generated content, and strategic integration of AI outputs, each imposing unique demands on writers' working memory and executive control (Liu et al., 2024; Nguyen et al., 2024; Jin et al., 2025). At the dispositional level, research has documented the role of learners' evaluative judgement and feedback literacy in mediating how writers interpret, act upon, and integrate AI-generated suggestions into their own work (Meng et al., 2026), as well as the importance of metacognitive regulation in sustaining productive engagement with AI tools (Yao and Fan, 2026a). Furthermore, learners' confidence in orchestrating the human-AI writing process, including prompt formulation, output verification, and ethical boundary management, has been shown to directly shape their persistence and emotional responses during AI-assisted composing (Yao and Fan, 2026b; Wang et al., 2025). Together, these lines of inquiry converge on a set of interrelated competencies that learners must marshal: the cognitive capacity to manage novel task demands, the evaluative skills to critically assess AI outputs, and the motivational resources to sustain engagement.

Collectively, these empirically validated constructs provide a vital taxonomy of the foundational competencies learners bring to GenAI-assisted writing—a map of what they know, can do, and believe at a given moment. Yet they remain largely static, cross-sectional conceptualizations that cannot explain why a learner's confidence, anxiety, or engagement surges or plummets across different writing tasks and social contexts. Writing is never a static event; it unfold across time, and emotional engagement fluctuates with task demands, interpersonal interactions, and evolving self-assessments. These dynamic processes, precisely what cross-sectional measures cannot capture, constitute the focus of the next section, which turns to the longitudinal, socioculturally grounded investigation of emotional engagement in GenAI-assisted writing.

### 2.2 Emotional engagement in GenAI-assisted writing

Engagement is a foundational concept in L2 research, denoting active learner involvement (Mercer, 2019) and serving as a key predictor of improved achievement, persistence, and retention (Bond et al., 2020; Kuh et al., 2008). Disengagement, by contrast, is associated with negative learning outcomes and cognitive development (Ma et al., 2015) and constitutes a known predictor of dropout at both secondary and tertiary levels (Bond et al., 2020; Finn and Zimmer, 2012). In EFL contexts, student engagement is commonly conceptualized as a tripartite framework encompassing behavioral, cognitive, and emotional dimensions (Fredricks et al., 2004; Zhang and Hyland, 2018). Behavioral engagement pertains to the quantity and quality of active participation, including rule obedience, voluntary involvement, interactional initiative, and task persistence (Reschly and Christenson, 2012; Shen et al., 2024). Cognitive engagement involves deliberate mental investment, characterized by selective and sustained attention to multiple resources in pursuit of learning goals (Reeve, 2012; Shen et al., 2024; Zhou et al., 2021). Emotional engagement manifests as learners' positive affective responses, i.e., positive dispositions toward learning materials, activities, peers, etc., during the learning process (Hiver et al., 2021; Shen et al., 2024).

Among the three engagement dimensions, emotional engagement has attracted growing scholarly attention, given its well-documented link to learning outcomes (Tyng et al., 2017). Positive emotions and attitudes, as indicators of emotional engagement (Ma et al., 2026; Yeung, 2025), may facilitate openness and deeper cognitive processing (Pitt and Norton, 2017), and emotionally engaged learners tend to persist with challenging tasks, employ effective learning strategies, and attain higher proficiency (Huo, 2022; Kohnke and Moorhouse, 2025). Negative emotions and attitudes, conversely, may signal emotional disengagement, triggering avoidance, superficial engagement, or resistance (Pitt and Norton, 2017). Such negative affect should not, however, be equated with disengagement in itself. Shen et al. (2026) showed that negative emotions do not uniformly undermine self-regulated learning: anxiety, for instance, negatively predicted some strategies (e.g., self-evaluation) but positively predicted others (e.g., persistence). Accordingly, emotional disengagement should be inferred not from negative valence alone but from the interplay of valence, attitude, and contextual embeddedness.

Unlike behavioral engagement, which is externally observable, and cognitive engagement, which involves mental effort and strategy use, emotional engagement captures learners' internal emotional responses that arise during learning (Fredricks et al., 2004). It is inherently more dynamic and context-dependent, fluctuating with task demands, interpersonal interactions, and environmental conditions (Pekrun and Linnenbrink-Garcia, 2012; Saeli and Rahmati, 2023; Xu and Zhang, 2025). This context-sensitivity is particularly pronounced in technology-rich environments, where reduced social cues and the absence of physical presence render emotional engagement both more critical and more vulnerable (Aladsani, 2022). In GenAI-assisted writing contexts, learners interact with artificial agents whose instantaneous, algorithm-driven responses may elicit unique emotional experiences ranging from satisfaction with immediate feedback to frustration over misinterpreted prompts, anxiety about skill atrophy, or alienation from machine-mediated authorship (Kohnke and Moorhouse, 2025; Yao and Liu, 2026). These affective responses differ qualitatively from those in conventional classrooms, highlighting the need for dedicated investigation of emotional engagement in GenAI-mediated writing environments.

In addition, extensive research has revealed the interconnectedness among the three dimensions of engagement, each capable of reinforcing or impeding the others (Koltovskaia, 2020; Ma et al., 2026; Mahfoodh, 2017; Shen et al., 2024; Shi, 2021; Yu et al., 2019). For example, Yu et al. (2019) found that negative emotions such as resistance tended to undermine behavioral and cognitive engagement. In contrast, Mahfoodh (2017) and Koltovskaia (2020) reported that negative affective responses, including frustration, disappointment, and distrust, could facilitate successful revision and active cognitive processing. Ma et al. (2026) further illustrated that cognitive engagement shaped behavioral and affective responses: low cognitive effort yielded generic feedback and reinforced skepticism, whereas deeper engagement elicited actionable feedback, facilitating revision and positive affect. Emotional engagement, in turn, influenced cognition—dissatisfaction often prompted iterative GenAI interaction, fostering deeper cognitive processing. These findings underscore the necessity of documenting behavioral and cognitive engagement when examining emotional engagement.

Recent empirical studies examining EFL learners' emotional engagement with GenAI feedback have revealed students' generally positive yet variable affective responses. Koltovskaia et al. (2024) observed emotional engagement among six Iranian graduate students, who preferred ChatGPT for paraphrasing to refine writing style. Similarly, Yan and Zhang (2024) found that four Chinese students' emotional engagement with ChatGPT feedback was predominantly positive, though behavioral engagement varied with language proficiency and technological competence. Besides, Xu et al. (2025) examined 29 postgraduate students' engagement with ChatGPT feedback and found moderate affective satisfaction during the revision process. Yeung (2025) further found that although engineering students generally showed positive emotions when engaging with GenAI feedback, negative feelings were observed when the feedback was perceived as general or ambiguous. More complex affective patterns emerge in Ma et al.'s (2026): most of six Chinese EFL students held mixed attitudes toward ChatGPT feedback—with one participant notably skeptical—and experienced emotions ranging from appreciation, disappointment, annoyance, and escalating dissatisfaction while processing GenAI feedback and revising. These studies indicate that learners' emotional responses to GenAI feedback are generally positive yet substantially heterogeneous, shaped by both individual differences and contextual factors in GenAI-mediated writing environments.

However, these empirical findings, while illuminating, point to three gaps in the literature. First, existing studies have largely focused on the revision stage, leaving emotional dynamics during pre-writing, drafting, and post-writing reflection underexplored. Second, longitudinal investigations into emotional dynamics throughout the writing process remain limited, leaving the evolution trajectory of emotional engagement over time insufficiently examined. Third, individual and contextual factors have been examined in isolation rather than as an interconnected system (Mao and Lee, 2022). Addressing these gaps requires a theoretical lens capable of capturing the dynamic, socially mediated nature of emotional engagement across the entire writing process.

### 2.3 A sociocultural framework for student engagement in writing

Sociocultural theory (SCT), originating from Vygotsky (1978), offers a productive lens to capture the complex, dynamic interplay between learners and their evolving writing contexts and explain how engagement fluctuates over time or across tasks, as it frames human learning as a socially mediated, context-embedded process. Mao and Lee (2022) operationalize Vygotsky's SCT principles into an analytical framework tailored to the context of L2 writing feedback, specifying both the nature of engagement and its underlying mechanisms (see Figure 1). In this framework, individual mediating factors include lived experiences (mindsets, beliefs, personal expectancies/values) and developmental competencies (cognitive skills, emotional regulation/control); environmental factors encompass specific learning tasks (writing task type, evaluation criteria of writing and revising), classroom setting (instructional focus, student-teacher/peer social relationships), and school environment (norms, policy). This framework is particularly applicable to technology-enhanced learning environments (Qian and Li, 2026), as it foregrounds the dialectical relationship between learners (individual) and their evolving contexts (environment). Accordingly, the present study adopts this framework as its analytical lens to examine the evolution of emotional engagement during a GenAI-assisted writing course.

**Figure 1:**
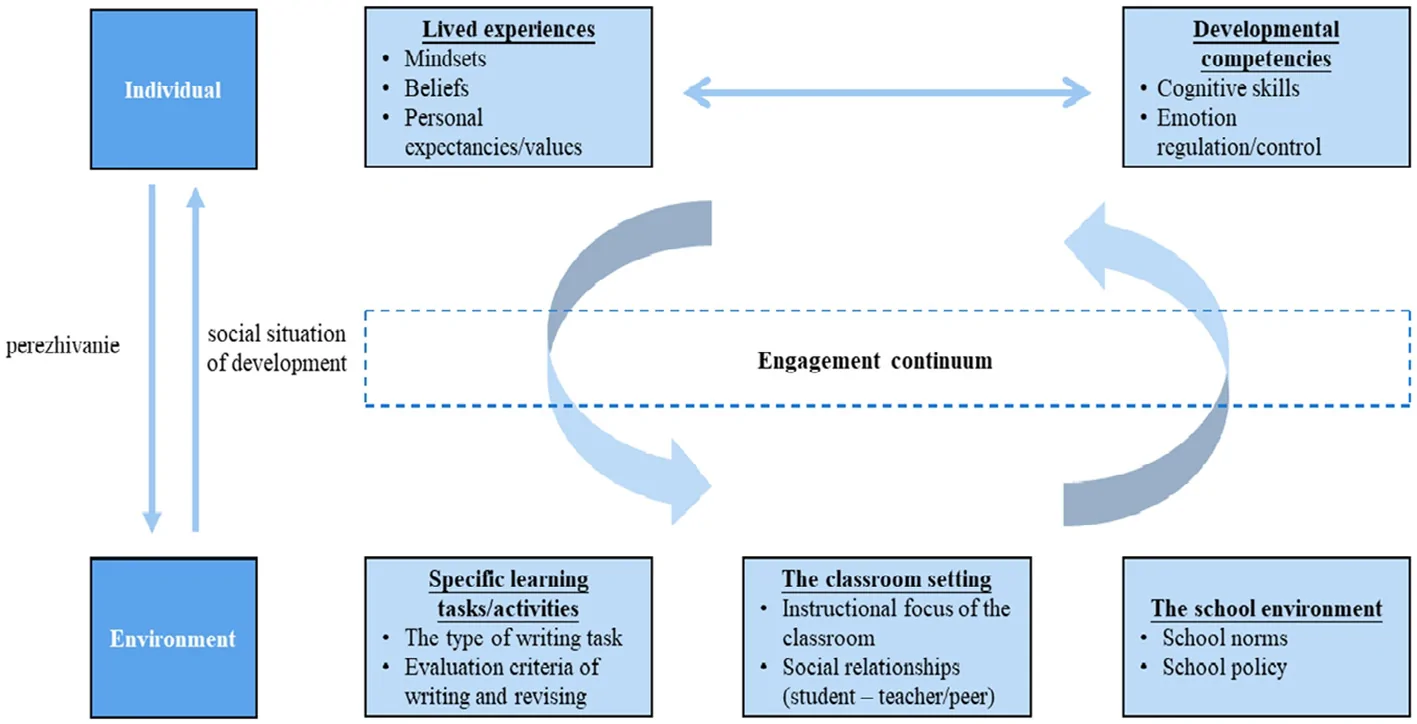
Sociocultural framework of L2 student engagement with written feedback (Mao and Lee, 2022).

The sociocultural perspective, through key constructs such as mediation, the zone of proximal development (ZPD), dynamic assessment, and perezhivanie, offers precise theoretical tools to explain the genesis and evolution of emotional engagement in GenAI-assisted writing. First, Mediation, the process by which humans use physical and symbolic tools to regulate their actions (Vygotsky, 1978), is not merely cognitive but deeply affective. In our context, GenAI, teachers, and peers act as mediational tools that do not just transmit information but regulate emotional states. For example, when GenAI persistently corrects errors, it acts as a “merciless mirror” that can mediate either frustration (if the learner interprets it as a threat) or motivation (if reframed as a growth opportunity).

Second, the Vygotskian notion of the ZPD—defined as the distance between what a learner can do independently and what they can achieve with guidance—directly links the quality of mediation to emotional engagement. Within the ZPD, mediation functions not merely as cognitive scaffolding but as an affective regulator: when GenAI or peer support is timely, contingent, and calibrated to the learner's emerging competence, it reduces the emotional threat of task difficulty and fosters a sense of competence. This supportive mediation sustains positive emotional engagement by making the task feel challenging yet manageable. Conversely, when mediation is absent, insufficient, or misaligned (e.g., GenAI provides opaque feedback), the learner is left to struggle beyond their current capacity. Thus, the ZPD provides a critical boundary condition for understanding why the same GenAI feature produces opposite emotional outcomes: the determining factor is not the task difficulty *per se*, but the adequacy and quality of the mediation that bridges the learner's current and potential levels of development.

Dynamic Assessment (DA) extends the ZPD concept by emphasizing graduated mediation—support that is continuously calibrated to the learner's emerging competence—and by diagnosing ability through responsiveness to mediation rather than static performance alone (Poehner and Lantolf, 2013). In GenAI-assisted writing, effective DA-informed tools would adjust their support dynamically across the composing process, providing scaffolding when needed and withdrawing it as competence develops.

Fourth, Perezhivanie, Vygotsky's concept of “lived experience,” is the linchpin that explains individual heterogeneity in emotional engagement. Perezhivanie is the prism through which environmental factors are refracted (Vygotsky, 1994), representing an indivisible unity of personal and situational characteristics (Mok, 2015). It explains why the same GenAI-assisted writing task may elicit fundamentally different emotional responses in different learners: each learner interprets, understands, and emotionally relates to a given situation differently (Vygotsky, 1994). It is this subjective refraction that determines the developmental influence of that situation (Mao and Lee, 2022; Mok, 2015). Crucially, perezhivanie is not static but dynamically evolves as learners develop and encounter new critical incidents, reshaping how they experience subsequent events (Mok, 2015). This dynamism provides perezhivanie with its explanatory power for longitudinal engagement research, offering a conceptual tool for understanding why different learners, despite sharing the same GenAI-assisted writing environment, developed radically different emotional trajectories.

In sum, SCT does not just describe what factors influence engagement (as listed in previous studies), but reveals how these factors interact dialectically across time, transforming static learner variables into a dynamic, developmental process. Guided by the sociocultural perspective in general and Mao and Lee's (2022) framework in specific, the present study addresses two questions that emerge from the above identified gaps in the literature:

- How does the emotional engagement of Chinese EFL learners evolve during a GenAI-assisted college writing course?
- What individual and environmental factors, as specified in Mao and Lee's (2022) sociocultural framework, contribute to the evolution of their emotional engagement in this context?

## 3 Materials and methods

This study adopted a multiple-case study approach to explore EFL learners' emotional dynamics in GenAI-assisted writing and the factors contributing to them, with validity enhanced through multi-source data integration (Yin, 2018).

### 3.1 Research context

This study was conducted within a 14-week general English writing course for first-year medical students at a Chinese university, marking the inaugural integration of GenAI tools into writing instruction and processes at this institution. The university mandates English as a compulsory subject for all non-English-major undergraduates. To support GenAI integration, the university has implemented multiple initiatives, including the development of an institutional GenAI platform, the creation of a GenAI tool (24H Intelligent Assistant) for faculty and students, the provision of training workshops to enhance instructors' GenAI literacy, and the facilitation of teaching teams in formulating course-specific GenAI use policies.

As detailed in Table 1, the course incorporated two GenAI training workshops (approximately 30 min each) covering usage guidelines, institutional policies, and prompting techniques. Additionally, nine weekly GenAI discussion sessions (Wang, 2024; see Supplementary Data Sheet 1.1) were conducted to facilitate peer sharing of GenAI experiences and address emerging questions. Writing tasks (see Supplementary Data Sheet 1.2) encompassed three genres: exposition (individual writing), feature article (group writing), and argumentation (individual writing). The writing processes are illustrated in Figure 2. During pre-writing, GenAI tools were employed for outlining across all three genres, with the feature article additionally requiring group data collection. First drafts of exposition and argumentation were handwritten in class individually without access to electronic devices; the group feature article, however, could be composed outside class for logistical considerations. The use of handwritten outlines was designed to foster independent content organization during prewriting and to preclude device dependence during the paper-based drafting stage. In post-writing, first drafts were revised using feedback from GenAI, peers, and teachers. Prompt templates were provided in the training workshops and via the 24H Intelligent Assistant (see Supplementary Data Sheet 1.2) to scaffold students' use of GenAI for brainstorming topics, gathering ideas, and seeking feedback. For all three tasks, students submitted first and final drafts alongside reflective reports and GenAI-student chatlogs. Regarding assessment, first drafts of exposition and argumentation constituted 50% of the respective task grades, with the remaining 50% allocated to final drafts; the feature article was assessed solely on the final draft (100%). Full grading rubrics are available in Supplementary Data Sheet 1.2.

**Table 1:** Weekly schedule of the GenAI-assisted writing course.

Week	Content
Week 1	Exposition writing skills; GenAI-assisted writing workshop 1-Getting started with GenAI
Week 2	Exposition in-class writing; GenAI-assisted writing workshop 2-Mastering prompt engineering
Week 3	Exposition writing feedback; Weekly GenAI Discussion-1
Week 4	Feature article writing skills-1; Weekly GenAI Discussion-2
Week 5	Feature article writing skills-2; Weekly GenAI Discussion-3
Week 6-7	Feature article writing skills-3; Weekly GenAI Discussion-4
Week 8-9	Feature article writing feedback; Peer review session; Weekly GenAI Discussion-5
Week 10	Argumentation writing skills-1; Weekly GenAI Discussion-6
Week 11	Argumentation writing skills-2; Weekly GenAI Discussion-7
Week 12	Argumentation in-class writing; Weekly GenAI Discussion-8
Week 13	Argumentation writing feedback; Peer review session; In-class revision (based on teacher + peer + GenAI feedback); Weekly GenAI Discussion-9
Week 14	Review

**Figure 2:**
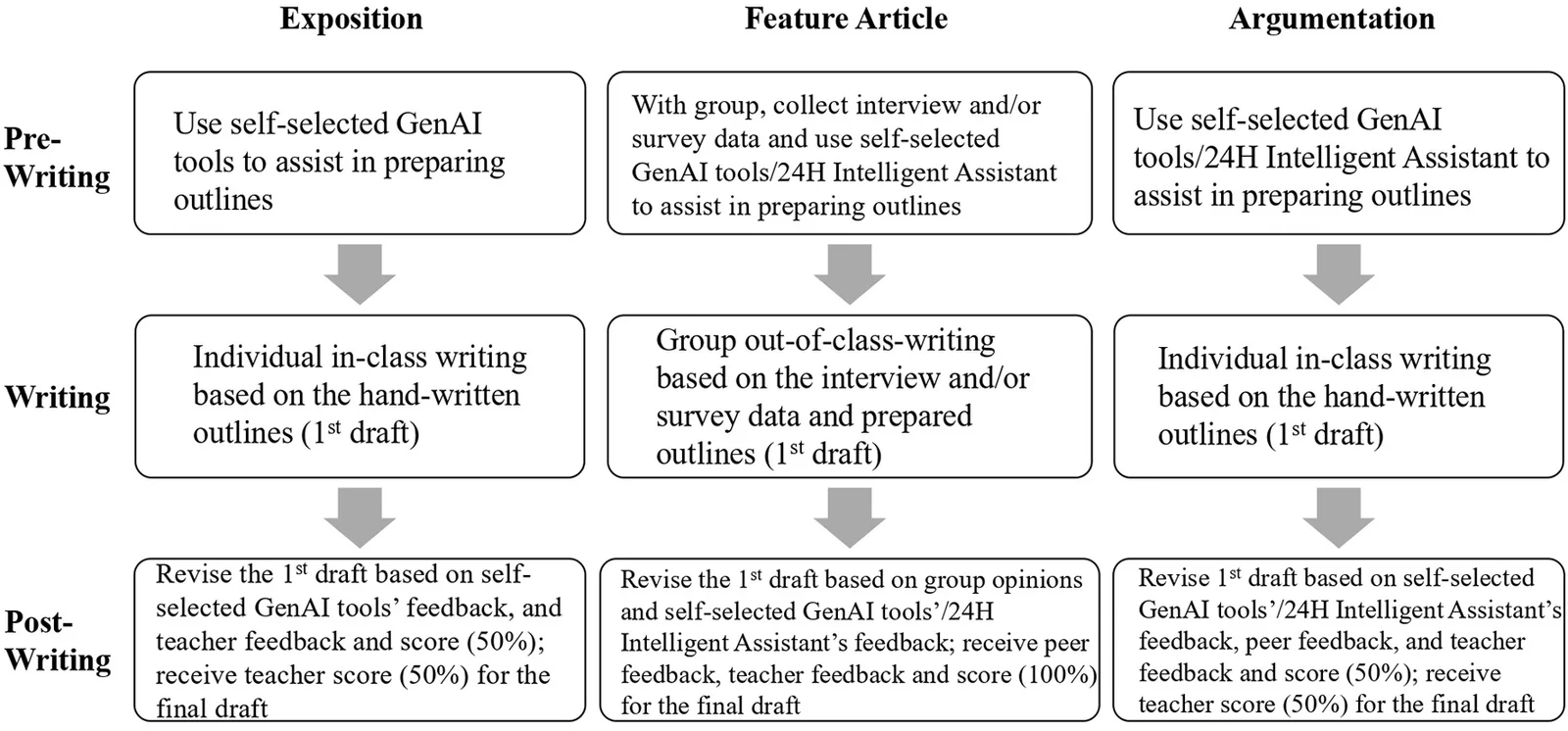
Writing processes of the three tasks.

### 3.2 Participants

Following a pre-course survey (*N* = 241) administered across seven classes that assessed students' prior GenAI experience and perceptions, 14 participants were purposively selected based on their willingness to participate, English proficiency levels, and pre-course GenAI use experience (Table 2). All participants were from the classes taught by the first and second authors. English proficiency was determined by students' English course scores from the previous semester (full score: 100, with 40% from class performance and 60% from the final examination covering listening, reading, vocabulary, and writing) and corroborated by teachers' evaluations. Participants were categorized into three proficiency groups: advanced (85–100), intermediate (70–84), and beginner (below 70). Regarding GenAI use experience, participants were classified as experienced (regular, weekly/bi-weekly use for EFL writing), rudimentary (infrequent, one-time to monthly use for EFL writing/non-English tasks), or novice (no prior use), based on self-reported frequency and purpose of GenAI use in the pre-course survey. Written informed consent was obtained from all participants, and all relevant ethical protocols were strictly followed. To mitigate evaluative pressure arising from the authors' dual roles as instructors and researchers, interviews were scheduled after task completion but before final grades were released. Participants were explicitly assured that their responses would not affect their course evaluation, and all prompts were framed in an open-ended, non-evaluative manner to encourage candid reporting.

**Table 2:** Background information of the 14 participants.

English proficiency^*^	Student (pseudonym)	Gender	Years of learning english	Prior GenAI use experience	Collected data
Advanced	**Leo (focal)**	Male	13	Experienced: regular GenAI use (weekly or biweekly) for EFL writing	**Three** rounds of interviews, informal chats, and reflective reports with **high** willingness to self-disclose; **complete** GenAI-student chatlogs
	Dora	Female	10	Rudimentary: infrequent GenAI use (one-time to monthly) for non-English tasks	Three rounds of interviews, informal chats, and reflective reports with moderate willingness to self-disclose; complete GenAI-student chatlogs
	Tina	Female	10	Rudimentary: infrequent GenAI use (one-time to monthly) for non-English tasks	Three rounds of interviews, informal chats, and reflective reports with moderate willingness to self-disclose; complete GenAI-student chatlogs
	Daniel	Male	10	Experienced: regular GenAI use (weekly or biweekly) for EFL writing	Two rounds of interviews and reflective reports with moderate willingness to self-disclose; complete GenAI-student chatlogs
	Thomas	Male	10	Rudimentary: infrequent GenAI use (one-time to monthly) for EFL writing	Two rounds of interviews and reflective reports with moderate willingness to self-disclose; complete GenAI-student chatlogs
Intermediate	**Grace (focal)**	Female	12	Experienced: regular GenAI use (weekly or biweekly) for EFL writing	**Three** rounds of interviews, informal chats, and reflective reports with **high** willingness to self-disclose; **complete** GenAI-student chatlogs
	Will	Male	10	Novice: no prior GenAI use	Three rounds of interviews, informal chats, and reflective reports with moderate willingness to self-disclose; incomplete GenAI-student chatlogs
	Olivia	Female	12	Rudimentary: infrequent GenAI use (one-time to monthly) for EFL writing	Two rounds of interviews and reflective reports with moderate willingness to self-disclose; complete GenAI-student chatlogs
	John	Male	10	Rudimentary: infrequent GenAI use (one-time to monthly) for non-English tasks	Two rounds of interviews and reflective reports with moderate willingness to self-disclose; complete GenAI-student chatlogs
Beginner	**Sarah (focal)**	Female	9	Rudimentary: infrequent GenAI use (one-time to monthly) for non-English tasks	**Three** rounds of interviews, informal chats, and reflective reports with **high** willingness to self-disclose; **complete** GenAI-student chatlogs
	Gavin	Male	12	Experienced: regular GenAI use (weekly or biweekly) for EFL writing	Three rounds of interviews, informal chats, and reflective reports with moderate willingness to self-disclose; incomplete GenAI-student chatlogs
	Amy	Female	10	Rudimentary: infrequent GenAI use (one-time to monthly) for non-English tasks	Three rounds of interviews, informal chats, and reflective reports with low willingness to self-disclose; incomplete GenAI-student chatlogs
	Robert	Male	10	Rudimentary: infrequent GenAI use (one-time to monthly) for EFL writing	Two rounds of interviews, informal chats, and reflective reports with low willingness to self-disclose; incomplete GenAI-student chatlogs
	Emma	Female	7	Experienced: regular GenAI use (weekly or biweekly) for EFL writing	Two rounds of interviews and reflective reports with high willingness to self-disclose; complete GenAI-student chatlogs

^*^Proficiency categories (advanced, intermediate, beginner) reflect local course-based groupings derived from students' English course scores and teacher evaluations, rather than standardized external benchmarks.

After data collection, three focal cases (Leo, Grace, and Sarah; see Table 2) were purposively selected for their varied proficiency levels, gender, GenAI use experience, and, crucially, data richness. All three completed three rounds of interviews, informal chats, and reflective reports with high willingness to self-disclose, and provided complete GenAI-student chatlogs and course artifacts. The selection also adopted Flyvbjerg's (2011) information-oriented strategies—paradigmatic, critical, maximally different, or extreme/deviant cases—appropriate for our in-depth multiple-case study. All three were 19-year-old Chinese L1 EFL learners from classes taught by the first author. Sarah's English learning background distinguished her from the other two focal students: she had studied Japanese throughout Senior 2 and 3 and taken Japanese as her foreign language examination subject for the College Entrance Examination.

### 3.3 Data collection

Semi-structured interviews with stimulated recall were conducted via Tencent Meeting with the three focal students after each of the three writing tasks, totaling 7 h and 5 min. Additional data sources included: researcher-participant informal chat histories documented throughout the semester; three essay outlines; first and final drafts across three tasks; reflective reports detailing students' GenAI use experiences and emotional responses (see Supplementary Data Sheet 1.3); GenAI-student chatlogs; research journals; and course syllabus that specifies GenAI policies, GenAI-assisted teaching/learning activities and assessment schema.

### 3.4 Data analysis

The interviews, researcher-participant informal chats, reflective reports, GenAI-student chatlogs, and course artifacts were examined via iterative analysis and triangulation (Srivastava and Hopwood, 2009) to investigate the focal students' emotional engagement evolution throughout GenAI-assisted writing processes. All dialogic data were transcribed verbatim via Tencent Meeting's built-in transcription tool and then translated into English using DeepSeek-V3. To ensure reliability, both the transcription and translation were cross-checked by two authors. To further verify semantic fidelity, particularly for emotion-laden expressions, back-translation was applied to a sample of the transcripts, and a Chinese teacher with 20 years of English teaching experience was invited to review the translated excerpts for accuracy and emotional nuance. This additional verification step was intended to mitigate the interpretive risk associated with machine translation of idiomatic and affectively rich speech.

To reduce potential interpretive bias from the first author's prior knowledge of students' performance, the second author participated in all stages of coding and theme development, offering an external perspective. Both authors independently reviewed a subset of the data, actively seeking disconfirming evidence and challenging initial assumptions to guard against confirmation bias. Disagreements in coding were systematically negotiated until consensus was reached. Triangulation across multiple data types further strengthened the validity of the findings beyond self-reported accounts. These procedures collectively enhanced the trustworthiness and credibility of the analysis.

To address RQ1, we used an analytical framework to examine learners' emotional, behavioral, and cognitive engagement (Table 3) adapted from existing operationalizations of engagement (Fredricks et al., 2004; Ma et al., 2026; Shen et al., 2024; Yeung, 2025). We then foregrounded and compared their emotional engagement in three GenAI-assisted writing tasks to track their emotional evolution. To ensure systematic cross-case and cross-stage comparisons, we adopted a qualitative analytical approach that focused on three dimensions of emotional evidence: the frequency of positive vs. negative emotion descriptors across multiple data sources (interview transcripts, reflective reports, and informal chat histories); the intensity of these emotional expressions, as inferred from lexical choices and repetition; and the contextual embeddedness of these emotions—whether they were sustained across tasks, recovered from setbacks, or eroded over time (see Table 4 for the evaluation framework). Drawing on these indicators, we identified the predominant emotional valence for each student at each stage and constructed individual emotional trajectories. This approach enabled us to synthesize each learner's emotional landscape into a coherent trajectory while maintaining the interpretive rigor appropriate to qualitative case study research. To address RQ2, we applied the sociocultural framework of L2 student engagement with written feedback (see Figure 1, Mao and Lee, 2022) to explain the underlying factors of student engagement evolution. We then conducted deductive thematic analysis about individual factors (lived experiences and developmental competencies) and environmental factors (specific learning tasks/activities, the classroom setting, and the school environment) (see Supplementary Table 1).

**Table 3:** The analytical framework of engagement for the study.

Engagement dimension	Operationalized definition
Emotional	Engagement: predominant positive emotions toward learning materials, activities, peers, etc., during the GenAI-assisted writing process; overall positive attitudes toward GenAI-assisted writing
	Disengagement: predominant negative emotions toward learning materials, activities, peers, etc., during the GenAI-assisted writing process; overall negative/indifferent attitudes toward GenAI-assisted writing
Behavioral	Engagement: sufficient quantity and quality of active participation in GenAI-assisted writing, e.g., obedience to writing rules, voluntary involvement in writing activities, interactional initiative with GenAI tools/peers, persistence in writing tasks, various revision operations, and proactive adjustment of GenAI use/writing strategies
	Disengagement: insufficient quantity and quality of active participation in GenAI-assisted writing, e.g., disobedience to writing rules, reluctance of voluntary involvement in writing activities, low interactional initiative with GenAI tools/peers, lack of persistence in writing tasks, limited revision operations, and limited adjustment in GenAI use/writing strategies
Cognitive	Engagement: sufficient mental investment and activities in GenAI-assisted writing, i.e., exhibiting deliberate, selective, and sustained attention to leverage multiple materials to achieve writing goals; demonstrating the depth of processing feedback; and employing cognitive/meta-cognitive operations to address feedback and facilitate revisions
	Disengagement: insufficient mental investment and activities in GenAI-assisted writing, i.e., exhibiting superficial, unfocused, or fleeting attention to leverage limited materials to achieve writing goals; demonstrating shallow processing of feedback and limited application of cognitive/meta-cognitive operations to address feedback and facilitate revisions

**Table 4:** The evaluation framework for emotional engagement and disengagement.

Engagement/Disengagement degree		Description (frequency + intensity + contextual embeddedness)	Example
Emotional engagement	Positive emotions markedly predominate	Positive emotion/attitude descriptors are consistently frequent across multiple data sources, with negative emotion/attitude descriptors being rare or virtually absent. Positive expressions are characterized by notably high intensity, as evidenced by strong lexical choices (e.g., “enthusiasm,” “a pretty positive cycle”) and frequent emphatic repetition. Positive emotions/attitudes remain stable across tasks, show resilience in the face of setbacks, or strengthen over time.	Leo: *GenAI has boosted my enthusiasm for learning English this semester. As I kept getting corrections from GenAI, I became more aware of my own weaknesses. And once I recognized those weaknesses, I felt motivated to address them. I'd say it created a pretty positive cycle*. (Informal chat 1)
	Positive emotions predominate	Positive emotion/attitude descriptors are more frequent than negative ones across multiple data sources, though negative expressions appear occasionally and remain clearly subordinate in both frequency and intensity. Positive expressions are moderately intense, as evidenced by generally affirmative lexical choices (e.g., “a sense of achievement”). Positive emotions/attitudes tend to be sustained across most tasks, with occasional fluctuations but an overall positive trend.	Leo: *I found this writing task a bit difficult…It was quite challenging for me. I still prefer writing on my own…During the interaction with GenAI, I feel a certain drive to think deeply about things I don't yet understand…when I do my own thinking, incorporate the errors GenAI points out, make revisions myself, and end up with a better piece of work, I actually feel a sense of achievement*. (Interview 1.2)
Emotional disengagement	Negative emotions predominate	Negative emotion/attitude descriptors are more frequent than positive ones across multiple data sources, though positive expressions appear occasionally and remain clearly subordinate in both frequency and intensity. Negative expressions are moderately intense, as evidenced by mildly critical or frustrated lexical choices (e.g., “insecure”). Negative emotions/attitudes tend to persist across tasks, with limited signs of recovery.	Grace: *Reading GenAI-generated text makes me feel really insecure. I've been learning English for so long, and it's not like I don't have access to resources, but I still can't reach that level…it makes me feel like my English is pretty poor*. (Interview 2.1)
	Negative emotions markedly predominate	Negative emotion/attitude descriptors are consistently frequent across multiple data sources, with positive emotion/attitude descriptors being rare or virtually absent. Negative expressions are characterized by notably high intensity, as evidenced by strong negative lexical choices (e.g., “really bad,” “quite low,” “really disappointed”) and frequent emphatic repetition. Negative emotions/attitudes are sustained across tasks, show little to no resilience, and may intensify over time.	Sarah: *The first time I wrote an exposition, I thought it was really bad, and later the score turned out to be quite low. I felt really disappointed. So this time when I had to write again, I just wanted to avoid it…Leaving the classroom to hide in the bathroom is also something I did back in middle school and high school*. (Interview 3.3)

## 4 Results

This section addresses the two research questions in turn. Section 4.1 charts the evolution of the three focal students' emotional engagement across the three writing tasks, with behavioral and cognitive engagement briefly noted as supplementary information to contextualize emotional responses. Section 4.2 then examines the individual and environmental factors that shaped these trajectories, explaining why the three cases followed qualitatively distinct paths.

### 4.1 How does the emotional engagement of Chinese EFL learners evolve during a GenAI-assisted college writing course?

#### 4.1.1 Leo: a positive trajectory of emotional engagement with temporary fluctuations

Leo grew up in a first-tier city, where his English learning journey began as early as the first grade of primary school. Supported by his well-educated parents, he developed a strong interest in English and a deep sense of its intrinsic value. He consistently excelled in English from primary school and won first prize in a provincial English speech contest during high school. In the previous semester, while peers struggled to grasp GenAI, Leo already utilized ChatGPT in English writing because it was “*more native-like*,” but he also tried to maintain his originality. His high English proficiency, early adoption of GenAI tools, and strong beliefs in native-like expression and authorship formed his initial social situation of development, positioning him as a confident and agentive learner.

In **Stage 1** (Exposition), Leo was excited when the writing class formally introduced GenAI assistance. Before writing, he independently selected the topic, crafted his thesis statement, used ChatGPT to gather examples, and drafted the outline by himself. This collaborative experience with GenAI left him thoroughly satisfied. He then completed his first draft in class smoothly. However, as Leo used GenAI to polish his draft, it acted like a merciless mirror, reflecting numerous grammatical errors and unnatural expressions (Figure 3). At that moment, this confident English learner experienced frustration and self-doubt for the first time: “*How could there be so many mistakes?... Now I'm even afraid to put pen to paper without GenAI”* (Interview 1.1). Nevertheless, upon receiving a teacher-assigned score (95) on his first draft that far exceeded his expectations, his belief in self-writing competence was gradually restored, reflecting his value on the student-teacher social relationship as a critical mediational resource. Overall, he summarized:

“*GenAI-assisted writing is like a process of sculpting, gradually perfecting it step by step... It gives me more motivation to write”* (Interview 1.1).

**Figure 3:**
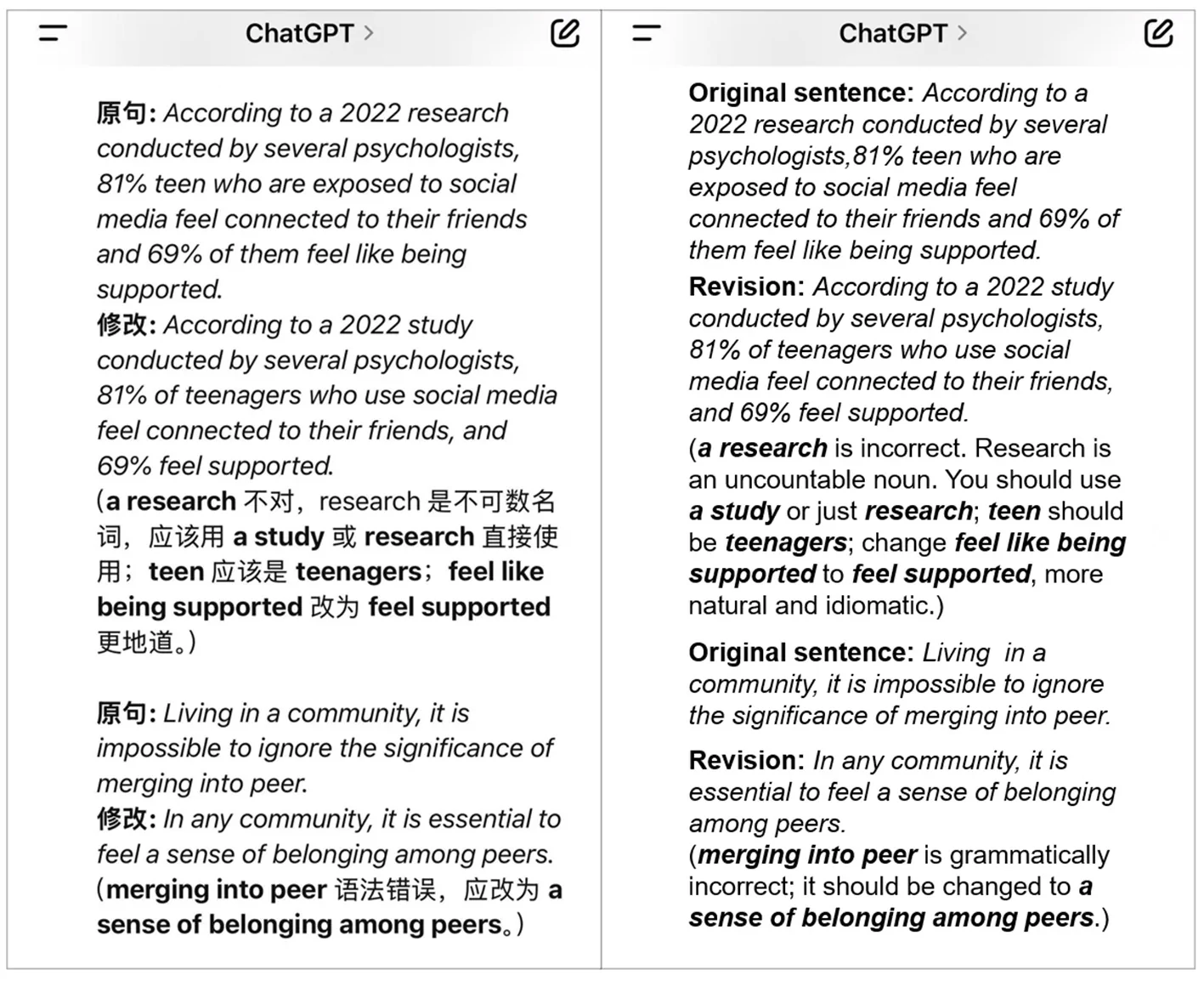
Excerpt from Leo's post-writing chat log with GenAI in Stage 1, showing error correction (original screenshot, left; translated version, right).

Although negative emotions (frustration, self-doubt) arose occasionally, Leo acknowledged experiencing more positive feelings (excitement, satisfaction, motivation) alongside a supportive attitude toward GenAI-assisted writing (“*It can benefit us for a lifetime,”* Reflective report 1), thereby demonstrating his emotional engagement in Stage 1. Furthermore, his sufficient quantity and quality of active participation in the writing process (e.g., interactional initiative with GenAI tools, persistence in writing tasks, various revision operations) and mental investment and cognitive skills in GenAI-assisted writing (e.g., employing cognitive/meta-cognitive operations to address feedback and facilitate revisions) also provided evidence of his behavioral and cognitive engagement.

In **Stage 2** (Group Feature Article), as group leader, Leo experienced complex emotional fluctuations. Before writing, he expressed interest in the group-GenAI collaboration, including creating the topic (*Dreaming in White Coats: What Drives Today's Medical Students?*) and conducting interviews based on the co-developed outlines. During writing, after group members completed their assigned paragraphs, Leo compiled and edited them into the first draft based on group discussion and his understanding, without using GenAI. He honestly admitted his dislike on this “*difficult and challenging”* collaborative writing experience (Interview 1.2), given the considerable responsibilities and workload he bore while balancing diverse ideas within a tight timeframe and an unfamiliar genre. After writing, he used GenAI to refine the essay's expression and structure, which evoked complex emotions: while he felt anxious about English writing because GenAI persistently challenged his writing knowledge, a sense of achievement remained predominant, accompanied by increased learning motivation. As he explained:

“*During the interaction with GenAI, I feel a certain drive to think deeply about things I don't yet understand…when I do my own thinking, incorporate the errors GenAI points out, make revisions myself, and end up with a better piece of work, I actually feel a sense of achievement” (Interview 1.2)*.

Receiving a score of 85 and teacher feedback on the final draft prompted Leo to engage in critical self-reflection that acknowledged the essay's limited depth while recognizing its structural strengths, illustrating that the student-teacher social relationship continued to serve as a valued and trusted mediational resource.

In this stage, responsible for coordination and management, Leo encountered heightened cognitive and behavioral demands, which, while fostering cognitive and behavioral engagement, also led to fluctuations in emotional engagement. His negative emotions (dislike and anxiety) were not merely individual reactions but emerged from the social situation of collaboration, peer dynamics, and task novelty. Nevertheless, his perezhivanie enabled him to sustain engagement despite these emotional challenges, alongside a positive yet increasingly critical stance toward GenAI-assisted writing (trusting GenAI for language advice but not structural revision).

In **Stage 3** (Argumentation), Leo returned to individual writing—his comfort zone. Remaining identical to Stage 1, the pre-writing procedures again elicited his satisfaction. The in-class writing session proceeded smoothly with his well-prepared outline. After writing, he used 24H Intelligent Assistant for the first time to obtain an essay score and feedback, but felt unused and turned to ChatGPT for further assistance. He then revised his first draft based on both GenAI and teacher feedback. Once again, he expressed his trust in teacher feedback (89) as the teacher had “*human thinking mode,”* but self-doubt, frustration and anxiety surfaced as GenAI continued to point out errors. Nevertheless, he ultimately experienced a sense of satisfaction, because “*GenAI helped me see my weaknesses more clearly and learn more”* (Interview 1.3). Rather than viewing anxiety as purely negative, Leo affirmed the learning motivation brought by it. He further expressed a positive attitude toward GenAI-assisted writing, accompanied by increased enthusiasm:

“*GenAI has boosted my enthusiasm for learning English this semester. As I kept getting corrections from GenAI, I became more aware of my own weaknesses. And once I recognized those weaknesses, I felt motivated to address them. I'd say it created a pretty positive cycle”* (Informal chat 1).

In this writing practice, Leo experienced more positive emotions (satisfaction, motivation, enthusiasm) than negative ones (self-doubt, frustration, anxiety) with a positive attitude toward GenAI-assisted writing, thereby achieving emotional engagement. Meanwhile, his behavioral engagement (actively and conscientiously revising compositions multiple times by integrating feedback from various sources; proactively comparing and experimenting with multiple GenAI tools) and cognitive engagement (actively mobilizing diverse learning resources through conscious, selective, and sustained focus to accomplish writing tasks; proactively employing a range of cognitive and metacognitive strategies) were also observable.

Corroborating this engagement profile, analysis of Leo's drafts across the three stages revealed a parallel textual evolution. Stage 1 revisions were primarily remedial, involving grammatical corrections and lexical substitutions (e.g., “makes a great difference” → “plays a pivotal role”), which reflected a surface-level, accuracy-focused engagement. By Stage 3, however, his revisions incorporated additional transitional phrases (e.g., “For example,” “while”) and summative sentences linking evidence to argument (e.g., “This heartbreaking incident highlights the consequences of inadequate sex education”), which may suggest a modest shift toward rhetorical awareness and deeper engagement, although genre and task format varied across stages.

#### 4.1.2 Grace: a trajectory from practical disengagement to critical engagement

Grace grew up as a migrant child in the rural outskirts of a first-tier city and did not receive systematic English instruction until the third grade of primary school. She attributed fluctuations in her previous English learning attitude and achievement to teacher quality, underscoring her emphasis on student–teacher relationships. As an intermediate-level EFL learner, she held weak belief in her self-writing competence. Before this semester, she had gained GenAI use experience and candidly admitted generating most previous essays with GenAI. This desire for efficient task completion reflects her utility value in English learning. Grace's intermediate proficiency, prior unguided GenAI use experience, utility value in English learning, weak belief in self-writing competence and firm belief in GenAI's usefulness formed her initial social situation of development, positioning her as a pragmatic, efficiency-driven learner.

In **Stage 1**, the writing assignment's multi-step requirements (drafts, reflections, GenAI chatlogs) disrupted Grace's efficiency-driven routine, eliciting initial annoyance. However, valuing the student-teacher social relationship, she complied with the instructions. During pre-writing, citing being “*too lazy to think”* (Interview 2.1), she directly prompted DeepSeek to explain the prompts for topic selection, then generated definitions and background for the introduction. She subsequently requested vocabulary, cohesive devices, and examples for body paragraphs, then added a conclusion and organized the material into an outline. This reliance on GenAI to bypass cognitive effort produced a sense of relaxation, yet she contributed few original ideas. During in-class writing, time constraints led her to deviate from the outline. After writing, to complete the reflective report intended to demonstrate critical thinking, she asked DeepSeek to generate two types of revisions of her first draft: a simple direct revision, and a revision based on a more elaborate template from the workshop and her personal strategies (Figure 4). She then composed her final draft and completed the reflective report based on a comparison of the two revisions. Despite receiving a high teacher score (92) for her first draft, GenAI's superior performance nonetheless triggered strong self-doubt and insecurity about her writing competence:

“*Reading GenAI-generated text makes me feel really insecure. I've been learning English for so long, and it's not like I don't have access to resources, but I still can't reach that level…it makes me feel like my English is pretty poor”* (Interview 2.1).

**Figure 4:**
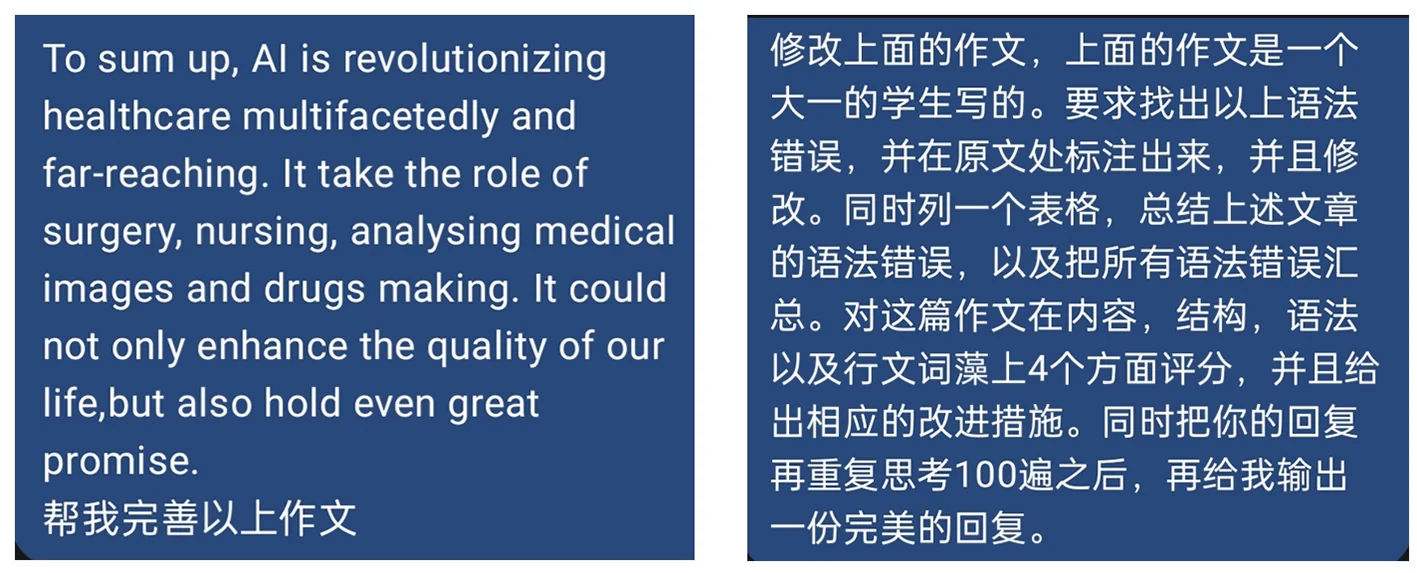
Grace's prompts to GenAI: a simple direct request (“Help me polish the essay,” left) vs. a more elaborate prompt (“Revise the essay written by a freshman. You should find out the grammar errors, highlight them in the text, and revise them. Meanwhile, generate a table to summarize the grammar errors. Grade the essay from four aspects: content, structure, grammar, and vocabulary; provide advice for improvement. And iterate the process 100 times to produce an optimal response,” right). The prompt text is reproduced from screenshots of the participant's GenAI chatlogs and translated into English. The phrase ‘iterate the process 100 times' reflects the participant's instruction to the model; it does not imply that the model actually performed 100 iterations or that outputs were verified.

Although Grace expressed a positive attitude toward GenAI-assisted writing, citing its efficiency in “*saving time and effort”* (Interview 2.1), her emotional disengagement was manifested predominantly by negative affect (annoyance, self-doubt, insecurity). Her sense of relaxation stemmed from using GenAI as a convenient tool to bypass mental investment and activities in GenAI-assisted writing, indicating cognitive disengagement. Nevertheless, her obedience to writing rules and proactive adjustment of GenAI use strategies demonstrated behavioral engagement.

In **Stage 2**, the environment expanded from individual-GenAI interaction to include peer mediation. Before writing, her group created the topic (*The Hidden Corner—Analyze the Call for Cleaners' Restrooms*) and collaboratively developed an outline. Grace participated in questionnaire design and interview conduct without GenAI involvement. During writing, she drafted detail paragraph 3, integrating questionnaire and interview findings to analyze growing public concern over the topic. She used Youdao (a translation tool) and Doubao (a GenAI tool) to translate Chinese materials into English, then reorganized and edited the information before submitting it to the group leader, who subsequently edited the first draft. Post-writing revision occurred through group discussion with Doubao's assistance. Grace expressed her feelings of liking and satisfaction toward collaborative writing:

“*I prefer group writing because it allows ideas to spark through discussion…We complemented each other, offered suggestions on each other's parts, and revised accordingly. I found it truly helpful”* (Interview 2.2).

The teacher score (90) also met her expectations. Despite anxiety, self-doubt and insecurity about her writing competence during GenAI interactions, she reported increased learning motivation owing to GenAI's convenience for self-learning. Although sufficient peer support reduced her frequency of GenAI use and reliance on it, she nonetheless maintained a sustained positive attitude toward GenAI-assisted writing.

On balance, she admitted that positive feelings far outweighed negative ones, as GenAI saved her time on English assignments, allowing more time for other activities and fostering emotional engagement. Additionally, her behavioral engagement (e.g., active participation in the writing process, interactional initiative with GenAI tools and peers) and cognitive engagement (e.g., leverage multiple materials like the output of GenAI and translation tools, class instructions, and peer feedback to achieve writing goals) were observable.

In **Stage 3**, before writing, Grace selected a topic and employed Doubao for brainstorming and outline generation, including an introduction, examples, and transitions. Applying a strategy learned from the workshops, she verified the validity of GenAI's example sources, a practice that brought her satisfaction. The in-class writing session proceeded smoothly using her well-prepared outline. After writing, her first revision focused on grammar and vocabulary based on feedback from GenAI (Doubao and 24H Intelligent Assistance). Surprisingly, she discovered grammatical errors in GenAI-generated text within the first draft. This critical incident shattered her previously held belief in GenAI's infallibility, creating a contradiction within her perezhivanie:

“*I trusted GenAI way too much with grammar, only to find out it could actually make grammatical errors. Unbelievable…I don't trust it anymore”* (Interview 2.3).

This breakdown of trust, however, served as a catalyst for transformation. Recognizing GenAI's limitations, she shifted strategy: using it to summarize collected examples, critically evaluating its Chinese output, and translating via Youdao to avoid errors. She applied this approach in the final revision, after incorporating feedback from the teacher and a more capable peer. Grace expressed her satisfaction on the teacher score (80) and appreciation for her peer's essay and feedback, acknowledging the peer as a learning model.

As in Stage 2, self-doubt and anxiety about her writing ability persisted but were confined to exam situations. Most of the time, she experienced predominantly positive feelings (satisfaction, relief, happiness) in GenAI-assisted writing and maintained a supportive attitude, as it reduced her study burden and increased learning motivation. Her emotional engagement thus became more resilient and differentiated, accompanied by behavioral engagement (e.g., adopting various revision operations, proactively adjusting GenAI use strategies) and cognitive engagement (e.g., employing cognitive and metacognitive operations to address feedback and facilitate revisions).

Grace's engagement profile was likewise reflected in her textual revisions. In the exposition, her revisions centered on clarifying the thesis and adding precision to existing examples (e.g., specifying “97.4% accuracy” for DeepMind Health), reflecting a growing attention to clarity and structure. By the argumentation task, however, she cited new empirical evidence in her essay, such as WHO (2023) and strengthened rebuttals with data, which may suggest a shift toward critical, evidence-based engagement. Notably, some grammatical errors persisted in her argumentation draft, suggesting that her attention was increasingly directed toward higher-order concerns such as evidence sourcing rather than surface accuracy, but this should be interpreted cautiously given the concurrent changes in genre and task format.

#### 4.1.3 Sarah: a trajectory from moderate disengagement to breakdown and intensified disengagement

Sarah grew up in a large city, where her English learning journey began in the third grade of primary school. However, the path soon turned rocky. In middle school, she found herself unable to persist with complex grammar, and her English performance declined sharply. By high school, a rigid and unsupportive teacher only deepened her resistance to English. In a pragmatic move to boost her College Entrance Examination scores, she switched to Japanese as her foreign language test subject in her second year, dedicating two years to learning Japanese while setting English aside. Upon entering university, she was unexpectedly required to take compulsory English course. Forced to resume classes despite a weak foundation, she admitted she did not perceive English as useful. Her encounters with GenAI had been minimal: only once, in the previous semester, for a non-English assignment. Sarah's beginner English proficiency, Japanese-as-a-foreign-language background, lack of perceived value in English learning, and limited prior GenAI experience formed her initial social situation of development, positioning her as a vulnerable and academically marginalized learner.

In **Stage 1**, Sarah encountered GenAI-assisted EFL writing for the first time, and her limited English proficiency soon became a barrier. She struggled to follow the English-medium instructions in the two workshops and, consequently, could not apply the prompting techniques taught but “*just used ‘generate' and ‘revise' over and over”* (Interview 3.1). After choosing a topic, she tasked Doubao with generating an outline and a full essay, repeatedly requesting revisions until the output no longer felt “*strange,”* and finally had it reorganize the essay into an outline. During in-class writing, she followed that outline to complete the introduction, conclusion, and topic sentences for two body paragraphs—all of which were detailed in the outline. However, the outline did not provide examples or explanations for the body, so she struggled to “make up” that content. Even with GenAI-generated outline, she felt anxious and overwhelmed while writing. Feeling “sorry” for the quality of her work, she left a note with her digital draft (Figure 5) explaining her background and hoping for the teacher's understanding: “*Sorry, teacher. I studied Japanese as my foreign language subject, so I don't yet understand English word classes or how to form proper sentences. I tried my best. I'm really sorry, teacher.”* Unfortunately, the teacher graded only the paper-based drafts and thus missed this honest confession. When Sarah received a score of 76, which she considered “*pretty low”* for her first draft (Interview 3.1), she felt strong disappointment and a loss of confidence, having failed to gain the teacher's “mercy”. In the post-writing revision, she “*basically just let GenAI do all the work,”* asking it to revise the “*strange parts”* until she could understand them, or else replaced them with simple sentences (Interview 3.1). A sense of insecurity about her writing competence emerged, as she noted, “*GenAI could think beyond me”* (Interview 3.1).

**Figure 5:**
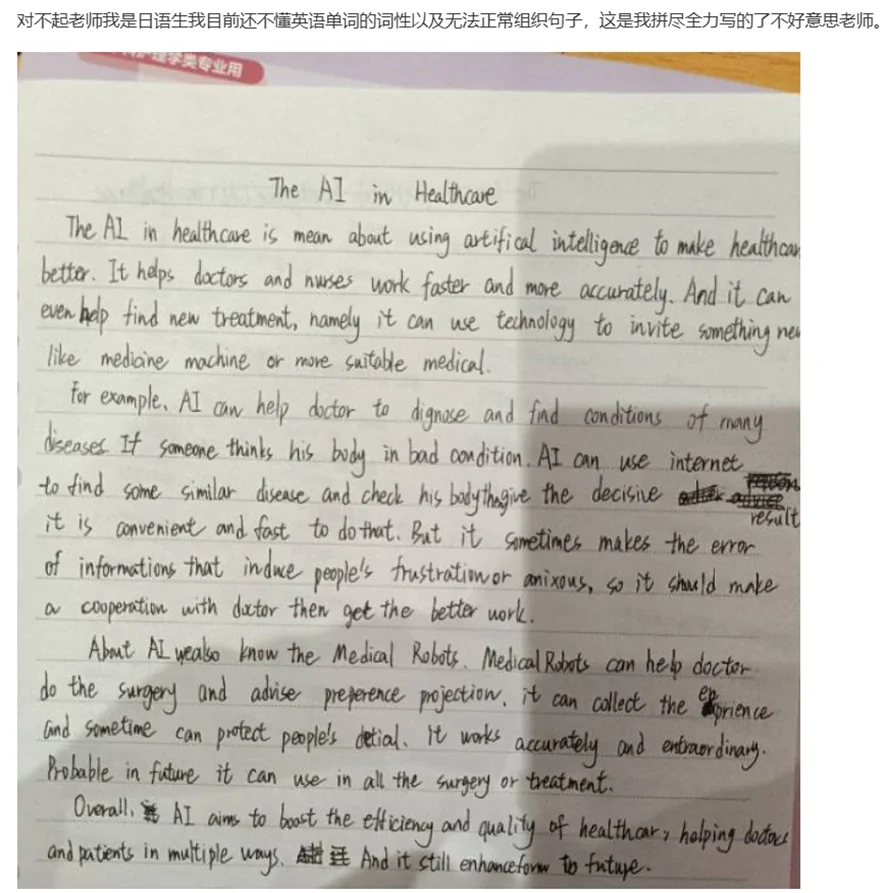
Sarah's note on her digital draft.

In this stage, Sarah expressed “*no feelings”* toward GenAI-assisted writing (Interview 3.1). She further acknowledged that it did not affect her learning motivation, as her motivation was consistently low and she studied only to pass the College English Test (CET) 4 and 6. Negative emotions (struggling, anxious, overwhelmed, sorry, disappointed, unconfident, insecure) predominated throughout, indicating her emotional disengagement. Her behavioral disengagement (e.g., limited revision operations, monotonous GenAI use strategies, few interaction turns with GenAI) and cognitive disengagement (e.g., relying on GenAI to generate outlines and most content, using GenAI to directly revise the entire text) were also observed.

**Stage 2** introduced a significant shift in Sarah's social situation of development through peer mediation. Her group created the topic (*Tired as a dog—Medical Students' Grueling Class Schedule*), and Sarah was assigned to conduct interviews. During writing, she took charge of one detail paragraph reporting the interview data. Instead of turning to GenAI, she strategically used a familiar translation tool, Baidu Input Method Translation (BIMT), while contributing content from the Chinese interviews she had conducted herself. As she explained:

“*Since it was a group task, I couldn't use my own awkward simple sentences…I organized the Chinese sentences myself, then used BIMT to translate them into English…This kind of writing made me feel more relaxed, because my group members had already provided a specific storyline, so I could just continue from where they left off. And since I conducted the interview myself, I knew exactly what to write”* (Interview 3.2).

After writing, she handed her paragraph to the group leader and, according to their task division, did not participate in the post-writing revision that involved GenAI. She felt satisfied with the teacher-assigned score (84).

Although her overall motivation for English learning remained low, Sarah reported a sense of relaxation and reduced writing anxiety during this process, indicating the emergence of emotional engagement. Moreover, under the social responsibility of teamwork, she demonstrated increased behavioral engagement (e.g., active participation in group collaboration, proactive selection of appropriate tools based on practical experience) and cognitive engagement (e.g., proactively mobilizing diverse learning resources such as translation tools, instructional content, self-collected data, and personal/group knowledge to complete the task), demonstrating that enriched social mediation can temporarily foster engagement even for deeply disengaged learners. Here, peer mediation may have brought the writing task within Sarah's ZPD, potentially enabling improved performance through structured scaffolding.

In **Stage 3**, Sarah experienced a profound emotional collapse. As in Stage 1, she chose the topic and then prepared an outline fully generated by DeepSeek. Yet when faced with an individual writing context similar to Stage 1 and an unfamiliar genre, she became overwhelmed by the fear of foreseeable failure. During in class writing, she physically fled the classroom. Her anxiety and lack of self-confidence escalated sharply. As she later explained:

“*For writing tasks like argumentation, where I'm given a topic and need to develop my own ideas, I often feel totally lost. Since I never wrote English essays in high school, I don't know how a normal person should write one. I feel like my openings or paragraphs must be wrong…The first time I wrote an exposition, I thought it was really bad, and later the score turned out to be quite low. I felt really disappointed. So this time when I had to write again, I just wanted to avoid it…Leaving the classroom to hide in the bathroom is also something I did back in middle school and high school”* (Interview 3.3).

Following this episode, the teacher met with Sarah privately to check on her wellbeing and discuss alternative ways to participate in the course, ensuring that she received appropriate support. Eventually, the teacher allowed her to submit the first draft after class. Since it became an out-of-class task, Sarah admitted that she used BIMT again to translate everything based on DeepSeek's outline. This time, her first draft received 78 points from the teacher, a score she considered fair given her level. For post-writing revision, she used feedback from both the teacher and DeepSeek to polish her essay, yet expressed a clear preference for teacher feedback, citing the need for “*human-level guidance”* suited to her learning context (Informal chat 3). She also declined to use the recommended tool, 24H Intelligent Assistant, for she was “*too lazy to explore”* (Informal chat 3).

Sarah remained indifferent to GenAI integration, viewing it as merely a tool and complaining it was more troublesome than BIMT. Overwhelmed by negative emotions (fear, anxiety, lack of self-confidence), her emotional disengagement intensified in this stage. This was accompanied by behavioral disengagement (e.g., disobedience of writing rules, no voluntary involvement in writing activities) and cognitive disengagement (e.g., limited cognitive/meta-cognitive efforts, shallow processing of feedback).

Sarah's case offers only limited textual corroboration. Although her exposition drafts displayed some surface-level error correction, the argumentation drafts, produced primarily through translation and GenAI tools, cannot reliably reflect her own textual development. This absence of interpretable textual evidence is a limitation of the data rather than a finding about her engagement; her trajectory therefore rests on self-report and observational data.

Taken together, the emotional engagement trajectories of three focal students are illustrated in Figure 6, based on qualitative coding of emotion descriptors in interview transcripts, reflective reports, and researcher-student informal chats. Leo exhibited a positive-resilient trajectory across three stages, despite temporary fluctuations in Stage 2. Grace followed a pragmatic-transformative path—from practical disengagement, through socially mediated positive affect, to critical engagement. Sarah displayed a vulnerable-contingent trajectory, moving from moderate disengagement and tentative, socially mediated positive affect to eventual breakdown and intensified disengagement. For Leo and Grace, textual evidence offered supplementary corroboration for these trajectories, revealing a gradual shift toward rhetorical awareness in Leo's revisions and a movement toward evidence-based argumentation in Grace's. In Sarah's case, interpretable textual evidence was unavailable.

**Figure 6:**
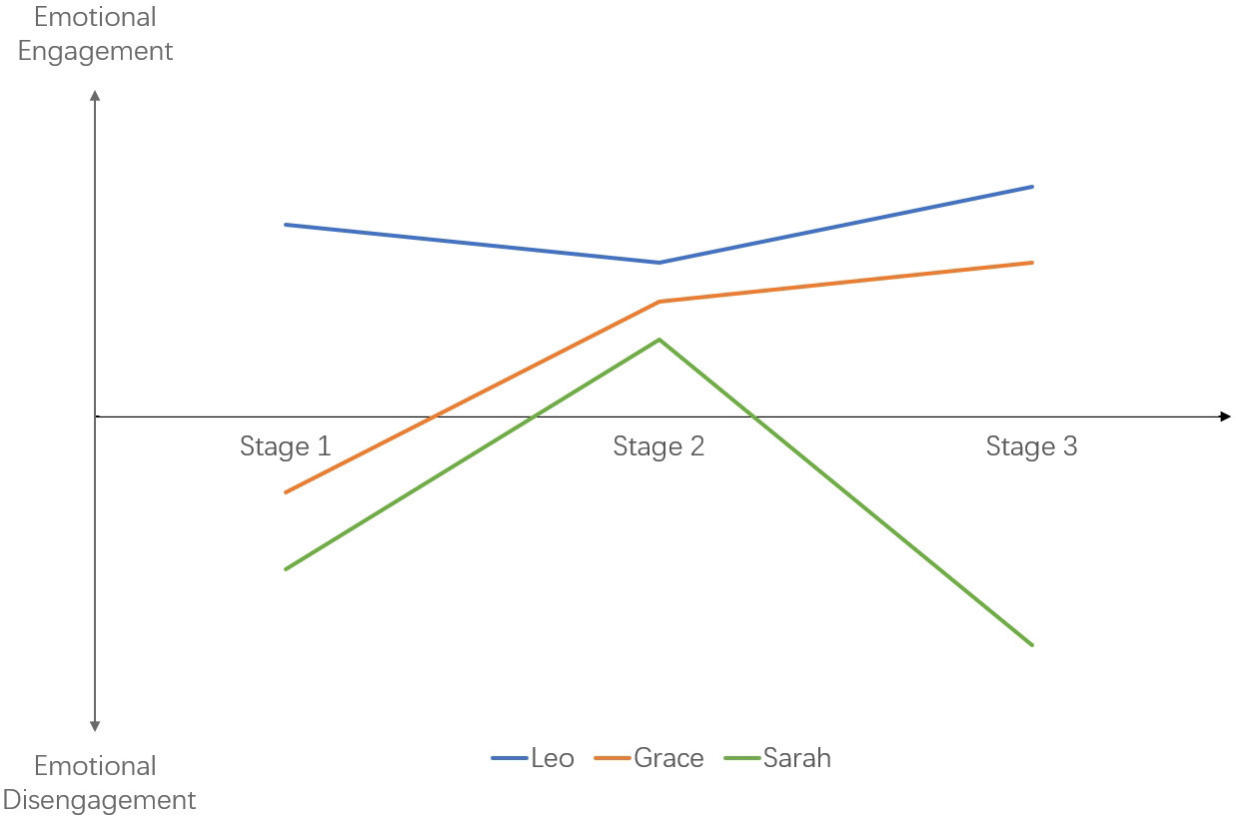
The emotional engagement trajectories of three focal students. The horizontal axis denotes the three sequential stages of the GenAI-assisted writing course; the vertical axis represents the relative valence of emotional engagement (ranging from disengagement to engagement), qualitatively inferred from the data rather than measured numerically.

It should be noted that, in Figure 6, each node's vertical position represents the relative valence of emotional engagement as qualitatively inferred from the data, rather than a numerically quantifiable measure. The degree of engagement at each stage was determined through systematic coding of three dimensions: the frequency of positive vs. negative emotion descriptors across multiple data sources; the intensity of these expressions as evidenced by lexical choices and repetition; and the contextual embeddedness of these emotions—whether they were sustained across tasks, recovered from setbacks, or eroded over time. For instance, Leo's consistently higher positioning reflects the predominance of positive emotion descriptors, his articulated sense of agency and achievement, and his demonstrated capacity to recover from temporary dips—all of which were recurrently evidenced across his three writing tasks. In contrast, Grace's trajectory shows a gradual upward trend, yet her Stage 3 positioning, while higher than Stage 2, does not reach Leo's level; this is because her emotional experience remained punctuated by persistent self-doubt and anxiety about her writing competence (see 4.1.2 for detailed evidence)—with these negative emotions primarily confined to exam situations. Similarly, Sarah's initial low positioning reflects the overwhelming prevalence of negative emotion descriptors and her expressed indifference toward GenAI-assisted writing; her Stage 2 improvement, while notable, was positioned only moderately higher due to the tentative and context-dependent nature of her positive affect, which was contingent on peer support rather than indicative of durable internal change (see 4.1.3). The trajectories thus represent a qualitative synthesis of each learner's emotional landscape across stages, capturing both the overall valence and the resilience or fragility of their emotional engagement over time.

### 4.2 What individual and environmental factors contribute to the evolution of their emotional engagement in this context?

Grounded in the sociocultural framework adapted from Mao and Lee (2022) (see Figure 1), this section analyzes how individual and environmental factors dialectically interacted to shape the three focal students' emotional engagement trajectories (Table 5).

**Table 5:** Factors contributing to the evolution of emotional engagement in three cases.

Case	Individual factors		Environmental factors			
	Lived experiences	Developmental competencies	Tasks/Activities	Classroom setting		School environment
				Instructional focuses	Social relationships	
Leo	• Growth mindset^*^ • Firm belief in GenAI's usefulness; fluctuating belief in self-writing competence • High expectancies for success; intrinsic value in English learning^*^	• High English proficiency • Rich cognitive skills^*^ • Adaptive emotion regulation strategies^*^ • Developing GenAI literacy^*^	• Tasks: individual exposition and argumentation writing (GenAI used in pre- and post-writing sessions); group feature article writing (GenAI used in out-of-class writing sessions; authentic student-collected data demanded) • Evaluation requirements: − Submissions: first & final drafts + reflections + GenAI chatlogs; − Assessment: exposition and argumentation: 50% respectively, for first & final drafts; feature article: 100% for Draft 2	• Essay development (exposition, feature article, argumentation) • Two GenAI-assisted writing training workshops • Nine weekly GenAI discussion sessions	• Positive student-teacher relationship • Positive student-peer relationship	• English as a compulsory subject for non-English-major undergraduates • Supportive policies on GenAI-teaching integration (developing an institutional GenAI platform and tool, providing training workshops for teachers, facilitating teaching teams in formulating course-specific GenAI use guidelines)
Grace	• Fixed mindset → Growth mindset^*^ • Firm belief in GenAI's usefulness; weak belief in self-writing competence; firm belief → weak belief in GenAI's infallibility • Fluctuating expectancies for success; efficiency-oriented utility value in English learning^*^	• Intermediate English proficiency • Limited → Developing cognitive skills^*^ • Maladaptive → Adaptive emotion regulation strategies^*^ • Developing GenAI literacy^*^			• Positive student-teacher relationship • Positive student-peer relationship	
Sarah	• Fixed mindset^*^ • Weak belief in GenAI's usefulness and ease of use; fragile belief in self-writing competence • Low expectancies for success; low utility value in English learning^*^	• Low English proficiency • Limited cognitive skills^*^ • Maladaptive emotion regulation strategies^*^ • Undeveloped GenAI literacy^*^			• Negative student-teacher relationship • Positive student-peer relationship in collaborative writing; negative student-peer relationship in peer feedback sessions	

^*^The key concepts outlined above are informed by the following body of literature: Eccles and Wigfield (2002), Koltovskaia et al. (2024), Li (2025), Li et al. (2022), Martínez-Priego et al. (2024), and Wang and Xue (2022).

#### 4.2.1 Individual factors

Leo maintained relatively stable individual factors across all three stages. His lived experiences—a growth mindset, intrinsic value in English learning, high expectancies for success, and a firm belief in authorship—enabled him to interpret GenAI's corrective feedback as an opportunity for growth rather than a threat. His developmental competencies included advanced English proficiency, which allowed him to critically evaluate GenAI output; well-developed cognitive skills for making informed decisions about GenAI suggestions; adaptive emotion regulation strategies that reframed frustration as a catalyst for growth; and evolving GenAI literacy—from experienced user in Stage 1 to strategic user in Stage 3 who confined GenAI to objective aspects (e.g., language) while relying on his own judgment for subjective aspects (e.g., structure, content).

Grace's individual factors underwent notable transformation. In Stage 1, her lived experiences were dominated by a fixed mindset, utility value in English learning (efficiency-oriented motivation), low expectancies for success, firm belief in GenAI's usefulness, and weak belief in her own writing competence. Her developmental competencies included intermediate English proficiency, limited cognitive skills (relying on GenAI to bypass effort), maladaptive emotion regulation, and experienced but unguided GenAI use. By Stage 3, peer mediation and a critical incident discovering GenAI's grammatical errors prompted a shift toward a growth mindset, weak belief in GenAI's infallibility, slightly higher expectancies for success, adaptive emotion regulation, developing cognitive skills, and evolved GenAI literacy as a critical, strategic user.

Sarah's individual factors remained largely maladaptive and unchanged. Her lived experiences—a fixed mindset, low utility value in English learning, low expectancies for success, weak belief in GenAI's usefulness and ease of use, and fragile belief in self-writing competence—rendered her highly vulnerable to negative emotional responses. Her developmental competencies—beginner English proficiency, limited cognitive skills, maladaptive emotion regulation (avoidance and rumination), and rudimentary GenAI literacy—functioned as barriers to accessing GenAI training, understanding GenAI output, and engaging meaningfully with the writing process.

#### 4.2.2 Environmental factors

Environmental factors operated at multiple levels in shaping each learner's trajectory.

Regarding Learning Tasks/Activities, individual writing favored Leo's positive affect but triggered breakdown for Sarah, while group writing alleviated anxiety for Grace and Sarah yet generated frustration for Leo due to leadership demands. The multi-step task requirements (drafts, reflections, chatlogs) drove Grace's initial annoyance but later promoted deeper engagement, while for Sarah they added to her sense of being overwhelmed. The evaluation criteria (50% for first draft, 50% for final draft) created performance pressure that proved particularly detrimental for Sarah.

Within the Classroom Setting, instructional focuses—the two GenAI training workshops and nine weekly discussion sessions—benefited Leo and Grace, who had the resources to understand and apply the content. For Sarah, the English-medium instruction functioned as an additional barrier. The prompt templates provided in the 24H Intelligent Assistant served as effective scaffolding for Grace but remained unused by Sarah, who never learned to apply such techniques.

Student-Teacher Relationships played a significant mediating role. Leo and Grace valued teacher feedback as a “human thinking mode” providing emotional support beyond what GenAI could offer. Sarah's case, however, illustrates the consequences of failed teacher mediation: her digital note—a plea for understanding given her Japanese-as-foreign-language background—was missed by the teacher, and the low score she received deepened her disappointment and academic insecurity.

Student-Peer Relationships also shaped engagement, though effects varied. For Grace and Sarah, peer support in group writing provided affective scaffolding that reduced anxiety. For Leo, peer collaboration introduced challenges and frustrations, though it also fostered cognitive and behavioral growth.

At the macro-level, the School Environment, including policies making English compulsory, created pressure for Sarah, which compounded her anxiety without adequate support. However, for Leo and Grace, the same requirements, combined with stronger individual resources, did not produce the same debilitating effect.

#### 4.2.3 The dialectical interaction between individual and environmental factors

A key finding is that emotional engagement emerges from the dialectical interaction between individual factors and environmental affordances, not from either factor in isolation. The same environmental feature produced different effects for different learners: group writing enhanced engagement for Grace and Sarah (peer scaffolding compensated for their perceived inadequacies) but diminished it for Leo (leadership demands challenged his autonomy). Similarly, GenAI training workshops benefited Leo and Grace but excluded Sarah due to her limited English proficiency, showing that environmental affordances are only as effective as learners' capacity to access them.

The three cases collectively illustrate a continuum of how individual-environment alignment shapes the durability of emotional engagement. Sarah's Stage 2 engagement proved fragile because peer mediation, though temporarily supportive, did not fundamentally restructure her underlying beliefs, fixed mindset, or maladaptive emotion regulation. When the supportive context was removed, her engagement collapsed. By contrast, Grace's trajectory illustrates how environmental affordances (peer support, critical incidents) created contradictions within her perezhivanie that prompted gradual transformation: from fixed to growth mindset, from maladaptive to adaptive regulation, from uncritical to critical GenAI literacy. Furthermore, Leo's case demonstrates how well-aligned individual and environmental factors can sustain positive engagement even when temporary challenges arise.

## 5 Discussion

This study investigates the evolution of Chinese EFL learners' emotional engagement during a GenAI-assisted college writing course and the factors contributing to this evolution. Through a longitudinal multiple-case study of three focal students within a 14-week course, we traced their emotional trajectories and identified three qualitatively distinct emotional engagement trajectories: positive-resilient (Leo), pragmatic-transformative (Grace), and vulnerable-contingent (Sarah), each shaped by the dynamic interplay between individual factors and contextual affordances. These findings extend current understandings of L2 student engagement with written feedback by reconceptualizing emotional engagement as a dynamic, socially situated phenomenon, refining the sociocultural framework with GenAI-specific competencies, and complementing foundational cross-sectional research by revealing the processes through which static resources are emotionally negotiated and socially mediated over time.

### 5.1 Emotional engagement as a dynamic, non-linear trajectory

A central finding is that emotional engagement in GenAI-assisted EFL writing evolves along dynamic, non-linear trajectories (see 4.1), resonating with research identifying it as dynamic and contextualized (Saeli and Rahmati, 2023; Xu and Zhang, 2025). Leo's trajectory illustrates this non-linearity: his temporary emotional dips did not signify disengagement, as his internal resources (growth mindset, intrinsic value, and adaptive emotion regulation) enabled him to reframe frustration and self-doubt as catalysts for growth. This supports Han and Hyland's (2019) argument that “negative emotions are not necessarily devastating” (p. 10) and aligns with Xu and Zhang's (2023) finding that “negative affective engagement catalyzes further cognitive and behavioral engagement” (p. 7). Grace's trajectory shows a different form of non-linearity: she experienced emotional disengagement in Stage 1, but her engagement was subsequently transformed through external mediation (peer support and a critical incident—discovering GenAI's grammatical errors—that prompted reassessment of her relationship with the tool). Sarah's trajectory, by contrast, moved in the opposite direction, escalating from moderate to intensified disengagement as limited support left little room for recovery. For Sarah, emotional engagement was highly contingent on continuous social mediation, and apparent engagement in supportive contexts did not indicate durable transformation of underlying perezhivanie. This extends Zheng and Yu's (2018) finding that low-proficiency students feel frustrated when unable to close the feedback loop, showing how such frustration can escalate into breakdown and withdrawal from in-class writing when compounded by absent teacher mediation and removed peer scaffolding.

Notably, what distinguished these trajectories was not the presence of negative emotions *per se*. Echoing Shen et al.'s (2026) demonstration that negative emotions like anxiety relate differentially to self-regulated learning, emotional disengagement in our data was identified only where negative emotions and attitudes predominated—operationalized as high frequency, high intensity, and persistence rather than recovery across tasks.

These three trajectories challenge universalist claims that GenAI-assisted language education uniformly leads to positive psycho-emotional outcomes and increases learners' engagement (cf. Ghafouri, 2024). Instead, our findings suggest that GenAI's affective impact is heterogeneous and contingent on learner profiles and contextual conditions—a point that resonates with recent Q-methodology research identifying distinct emotional profiles among students using ChatGPT feedback (Yao and Liu, 2026). The sources of this heterogeneity and the processes through which GenAI's affective impact is mediated are examined in the next section.

### 5.2 Factors contributing to the evolution of emotional engagement

Mao and Lee's (2022) framework identifies lived experiences (mindsets, beliefs, personal expectancies/values) and developmental competencies (cognitive skills, emotional regulation/control) as key individual mediating factors. Our findings corroborate this while suggesting two important additions specific to GenAI-assisted writing: English proficiency and GenAI literacy (see 4.2.1 for detailed individual factor analysis).

English Proficiency emerged as a critical mediator shaping learners' capacity for emotional engagement. Leo's advanced proficiency enabled critical evaluation and sustained dialogue with GenAI, while Sarah's beginner proficiency barred her from GenAI training workshops and meaningful tool use. The English-medium workshops may have imposed extraneous cognitive load (Sweller, 1988) on Sarah, which spilled over into the affective domain, deepening her fear of failure and manifesting as overwhelming anxiety and withdrawal from in-class writing. This aligns with previous research demonstrating that language proficiency mediates the processes and products of student engagement (Buckley, 2018; Cheng and Liu, 2022; Kahu, 2013) and that low proficiency impedes learners' handling of feedback and revision (Pan et al., 2023; Zheng and Yu, 2018). Our study extends this literature by showing how proficiency interacts with GenAI-specific demands: low-proficiency learners are doubly disadvantaged, struggling with both the writing task and the linguistic and cognitive demands of effective GenAI use.

GenAI Literacy, encompassing prompt engineering, critical evaluation of GenAI output, adaptive strategy use, and selective tool application (Li, 2025), emerged as another critical developmental competency. Leo evolved from an experienced to a strategic user who confined GenAI to objective aspects (e.g., language) while reserving judgment on subjective aspects (e.g., structure, content); Grace shifted from an unguided heavy user to a critical, strategic user who verified GenAI's sources and combined tools strategically; Sarah's literacy remained rudimentary throughout. These differential trajectories suggest a potentially useful complementarity between cross-sectional measurement and longitudinal process data: while validated scales (Yao and Fan, 2026a) can identify the dimensional structure of literacy, process-oriented studies may reveal how these dimensions are dynamically negotiated through social interaction. Grace's critical evaluation skills, for instance, were activated only after peer modeling and a critical incident (discovering GenAI's grammatical errors) prompted reassessment of her relationship with the tool, whereas Sarah, in the same collaborative environment, showed no lasting development. As discussed below, this pattern appears to reflect a mismatch between the mediation provided and Sarah's developmental readiness within the ZPD, suggesting that the developmental mechanisms underlying literacy acquisition may be particularly sensitive to social mediation. GenAI literacy thus appears not as a set of static competencies but as a dynamically constructed resource shaped by both individual capacity and the availability of socially accessible instruction.

Perezhivanie, Vygotsky's concept of lived experience as the indivisible unity through which environmental factors are refracted (Vygotsky, 1994; Mok, 2015), provides a unifying lens for understanding how the same GenAI-assisted writing environment produced divergent emotional trajectories. Leo's perezhivanie was not simply a collection of positive traits; rather, his growth mindset, intrinsic value in English learning, and firm belief in authorship constituted a subjective prism through which GenAI's persistent error corrections were refracted as opportunities for growth rather than threats to competence. Grace's perezhivanie underwent transformation: initially dominated by utility value and efficiency-driven motives, her lived experience was gradually restructured through peer mediation and a critical incident, which reframed her relationship with the tool from one of uncritical reliance to critical engagement. Sarah's perezhivanie, by contrast, was rooted in academic insecurity and a fixed mindset shaped by years of perceived failure in English learning. This prism refracted the same environmental affordances (GenAI feedback, teacher scores, writing tasks) as confirmations of inadequacy, deepening her vulnerability and precipitating intensified disengagement that manifested in part as withdrawal from in-class writing. Very importantly, these cases illustrate that perezhivanie is not fixed but can evolve, though evolution requires not just exposure to new tools but fundamental restructuring of lived experience (Xu and Zhang, 2023, 2025). Emotion regulation strategies further differentiated the three cases (see 4.2.1), aligning with research that emotion regulation competence is crucial for sustaining engagement and that maladaptive regulation can precipitate disengagement across dimensions (Xu and Zhang, 2023).

Regarding environmental factors (detailed in 4.2.2), our findings reveal that task type significantly shapes emotional engagement, a dimension underexplored in feedback research which has largely focused on writing genres (Qian and Li, 2026). The effects were not uniform: individual writing fostered positive affect for Leo but triggered breakdown for Sarah; group writing alleviated anxiety for Grace and Sarah yet generated frustration for Leo due to leadership demands. These contrasting responses indicate that emotional consequences are not inherent to task types but arise from the interaction between task demands and learner profiles, consistent with evidence that peer feedback can both enhance and complicate engagement depending on interactional dynamics (Li and Qian, 2024). Similarly, GenAI training workshops benefited Leo and Grace but excluded Sarah due to her limited proficiency, showing that environmental affordances are only as effective as learners' capacity to access them. The multi-step task requirements drove Grace's initial annoyance but later promoted deeper engagement by making her GenAI use visible and accountable, suggesting that task design mediates both what students do and how they feel.

Student-teacher and student-peer relationships (see 4.2.2) further shaped engagement through direct affective mediation. Leo and Grace valued teacher feedback as a “human thinking mode” providing support beyond GenAI; Sarah's case illustrates the consequences of failed teacher mediation: her unacknowledged plea for understanding deepened her disappointment and academic insecurity, echoing research that trusting teacher-student relationships are crucial for revision behaviors and feedback use (Lee and Schallert, 2008; Henry and Thorsen, 2018). Peer support provided affective scaffolding for Grace and Sarah, aligning with research that peer relationships foster motivation and engagement (Wentzel et al., 2017), yet Sarah's engagement in Stage 2 proved fragile, collapsing when peer support was removed, revealing the contingency of peer-mediated engagement for vulnerable learners.

The notion of ZPD offers a useful interpretive lens for understanding the differential outcomes observed in our three cases. The critical variable is whether GenAI, teacher, or peer mediation is calibrated to the learner's emerging competence. When well-attuned, mediation (e.g., GenAI training workshops) may reduce anxiety and foster a sense of control, supporting emotional engagement. When opaque or misaligned, as appears to have been the case for Sarah whose limited English proficiency and minimal prior GenAI experience left her struggling beyond her ZPD, the same mediation may instead generate cognitive overload and contribute to emotional disengagement. This interpretation suggests that GenAI-assisted writing might be productively understood as a dialogic ecosystem in which the quality of mediation, rather than its mere availability, plays a significant role in shaping learners' emotional trajectories. At the same time, this remains a theoretically informed interpretation that would benefit from further empirical investigation.

The above analysis, together with what 4.2.3 elaborates, indicates a key theoretical insight: emotional engagement emerges from the dialectical interaction between individual factors and environmental affordances, not from either factor in isolation (Vygotsky, 1994). This explains why the same task produced different effects across learners: peer scaffolding compensated for perceived inadequacies in Grace and Sarah, while for Leo it introduced autonomy-challenging demands. Crucially, Sarah's Stage 2 engagement proved fragile because peer mediation, though temporarily supportive, did not fundamentally restructure her underlying beliefs, fixed mindset, or maladaptive emotion regulation. Therefore, when the supportive context was removed, her engagement collapsed. This pattern tentatively suggests that durable emotional engagement requires not only contextual support but also transformation of learners' internal worlds, a process that appears more likely to occur when environmental scaffolding is accompanied by opportunities for restructuring underlying beliefs and emotion regulation strategies (cf. Han, 2017).

Our findings also appear to be consistent with prior research documenting the interconnectedness of emotional, behavioral, and cognitive engagement (Fan and Xu, 2020; Han and Hyland, 2015; Shen et al., 2024; Shi, 2021), yet reveal that this interplay takes qualitatively different forms across learner profiles (see 4.1 for behavioral/cognitive engagement evidence). Leo exemplified a positive spiral where emotional engagement catalyzed behavioral and cognitive investment, which in turn produced successful outcomes that reinforced positive affect (Shen et al., 2024). Grace demonstrated behavioral engagement despite emotional and cognitive disengagement in Stage 1, echoing Shi's (2021) observation of mismatched engagement dimensions. However, her trajectory shows how behavioral engagement can, over time, lead to emotional and cognitive transformation when mediated by critical incidents and social support. Sarah's case revealed the most problematic pattern—predominant negative emotions precipitated disengagement across all dimensions, consistent with Yu et al.'s (2019) finding that negative affective engagement can result in lack of behavioral and cognitive engagement.

### 5.3 An adapted sociocultural framework of EFL student engagement with GenAI-assisted writing

Based on the empirical findings of this study, we tentatively propose an adapted sociocultural framework that extends Mao and Lee's (2022) original model to engagement with GenAI-assisted EFL writing process (Figure 7). This adaptation should be viewed as a theoretically informed synthesis of our case data, offering one possible lens for interpreting the observed emotional trajectories rather than a definitive model of GenAI-assisted writing engagement. Three key refinements are suggested. First, we add English proficiency and GenAI literacy as developmental competencies shaping learners' capacity to engage with GenAI tools. While these competencies have been identified and operationalized in recent measurement research (e.g., Jin et al., 2025; Yao and Fan, 2026a,b), our longitudinal data suggest that they function dynamically rather than statically—their development appears to be mediated by social interaction and task experience (cf. Jin et al., 2025; Wang et al., 2025). Second, we embed GenAI-related elements across environmental levels: GenAI-assisted tasks and feedback, GenAI training and peer discussions, and institutional GenAI policies. Third, we foreground the dynamic interplay among emotional, behavioral, and cognitive engagement, treating emotional engagement as a fluid state that interacts with the other dimensions over time. While offering a more comprehensive and dynamic lens for understanding how EFL learners' emotional engagement evolves in technology-mediated writing contexts, this adapted framework would benefit from further empirical testing in diverse contexts to assess its broader applicability.

**Figure 7:**
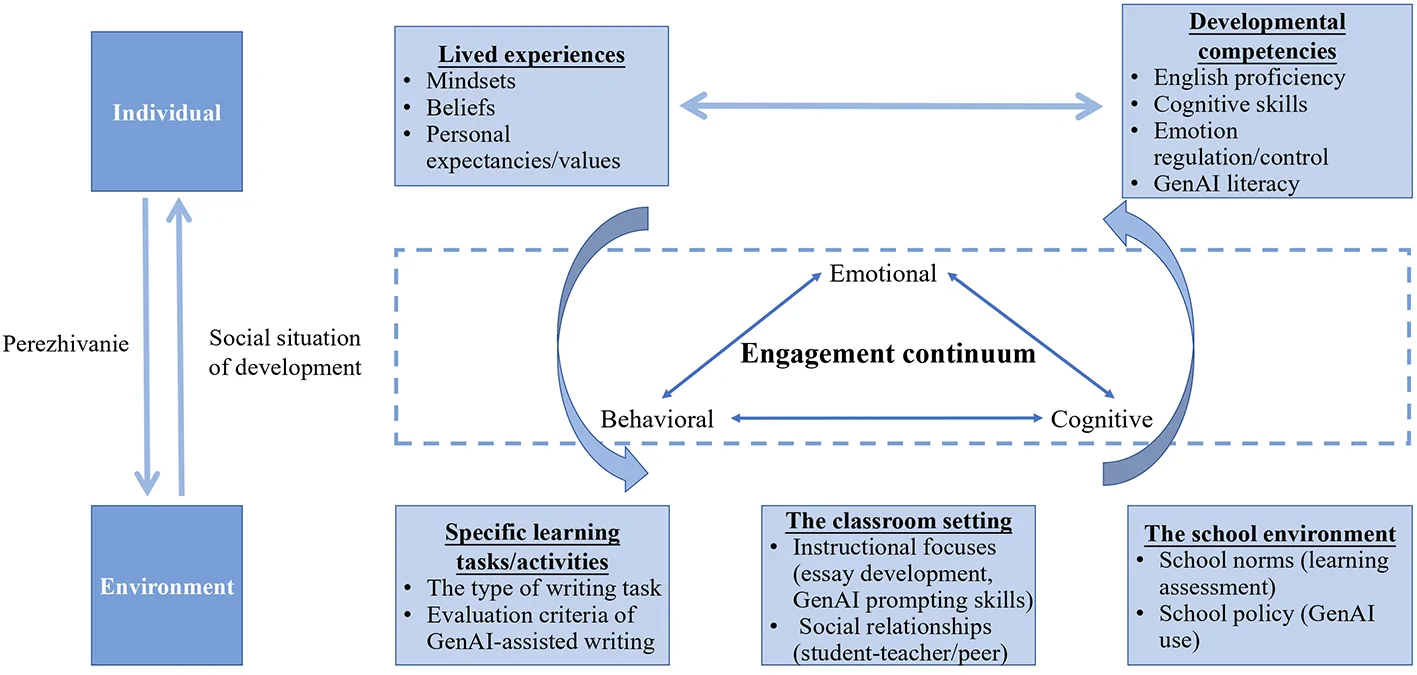
Sociocultural framework of EFL student engagement with GenAI-assisted writing [adapted from Mao and Lee (2022)].

## 6 Conclusion

This longitudinal multiple-case study traced the evolution of emotional engagement in GenAI-assisted EFL writing among three Chinese university students, revealing three qualitatively distinct trajectories—positive-resilient, pragmatic-transformative, and vulnerable-contingent—shaped by the dialectical interplay between individual factors (especially English proficiency, GenAI literacy, mindsets, beliefs, and emotion regulation) and environmental factors (task design, peer and teacher mediation, instruction, and institutional policies).

The study offers tentative theoretical contributions to understanding EFL students' emotional engagement in GenAI-assisted writing. First, it suggests an extension to Mao and Lee's (2022) sociocultural framework by (a) identifying English proficiency and GenAI literacy as potentially important developmental competencies that may condition learners' capacity to access, critically evaluate, and strategically leverage GenAI tools; (b) situating GenAI-related factors across task/activity, classroom, and school levels, thereby capturing contextual affordances and constraints distinct from conventional or earlier technology-enhanced writing; and (c) integrating the tripartite engagement model (Fredricks et al., 2004) to foreground emotional engagement as a fluid, dynamic state that interacts with behavioral and cognitive dimensions over time. Second, by revealing heterogeneous emotional trajectories and offering interpretive accounts of their emergence, the study offers a qualified challenge to universalist claims (cf. Ghafouri, 2024) and suggests a refinement of sociocultural theory to account for how human and non-human mediators may be refracted through learner's perezhivanie, producing divergent outcomes from ostensibly identical instructional conditions. This interpretation remains provisional pending replication and extension with larger and more diverse samples.

These findings yield several pedagogical implications. Differentiated support based on learner profiles is essential: advanced learners benefit from agency-preserving challenges; pragmatic learners require transformation of GenAI use from cognitive relief to genuine learning through critical incidents and peer mediation; vulnerable learners need affective scaffolding alongside foundational linguistic and GenAI literacy development. Explicit GenAI literacy instruction (Li, 2025; Tian and Xiang, 2026) should be accessible to all learners, differentiated by proficiency, and scaffolded for lower-proficiency students. Peer mediation can provide powerful affective scaffolding if accompanied by efforts to transform underlying beliefs and emotion regulation strategies. Teacher affective mediation remains irreplaceable, especially for vulnerable learners who may not voice their needs directly. Task design should balance individual and collaborative writing, attending to cognitive, linguistic, and affective demands. Finally, institutional support—clear policies, teacher training, and attention to how high-stakes requirements disproportionately affect vulnerable learners—is essential for sustainable and equitable GenAI integration.

Several limitations warrant acknowledgment. First, the small number of cases limits generalizability, although information-oriented selection of paradigmatic, critical, and extreme cases enhances transferability (Flyvbjerg, 2011). Second, the single-institution context may limit transferability to other cultural or disciplinary settings. Third, the study relied heavily on self-report data and researcher interpretation, though triangulation across multiple data sources enhanced validity. Fourth, the 14-week timeframe, while longer than most feedback engagement studies, may not capture long-term sustainability of emotional engagement patterns. Fifth, genre, individual/group format, and timing varied together across the three tasks, limiting attribution of observed changes to development over time. Finally, GenAI-assisted translation, despite verification procedures, may entail subtle affective nuance loss, particularly for idiomatic and emotionally charged expressions.

Future research should address these limitations through larger-scale, mixed-methods studies that examine emotional engagement across diverse institutional and cultural contexts. Longitudinal research tracking students across multiple semesters could reveal whether the trajectories identified in this study are sustained or transformed over longer timeframes, and could also enable closer examination of how emotional engagement influences writing development in the long run—a relationship that the present study was not designed to capture. In addition, research that separates the confounding influences of genre, task format, and time is also needed, so that developmental changes in emotional engagement and writing can be more precisely identified. Finally, to further mitigate the risk of affective nuance loss associated with GenAI-assisted translation, future studies could consider independent bilingual expert review of the full qualitative data corpus.

## Data Availability

The raw data supporting the conclusions of this article will be made available by the authors, without undue reservation.

## Footnotes

1 **Note on Terminology**: Throughout this article, we use “GenAI” as the primary term for generative artificial intelligence. However, when discussing studies that use “AI” to refer to the same concept (e.g., Jin et al., 2025; Yao and Fan, 2026a,b), we adopt “AI” for consistency and brevity.
