# Supplementary material for: Engagement or disengagement: understanding the emotional dynamics of EFL learners in GenAI-assisted writing from the sociocultural perspective
Source: Front Psychol. 2026 Oct 21;17:1934782. doi: 10.3389/fpsyg.2026.1934782 (PMC13731371; doi:10.3389/fpsyg.2026.1934782)
Supplement: Supplementary file 1 [file Supplementary_file_1.docx]

Supplementary Material

# 1 Supplementary Data

## 1.1 Weekly GenAI Discussion Directions

Weekly AI Lab: Try, Share, Grow

1.1.1 Try: Explore various AI tools throughout the semester to enhance your English learning, especially writing. Take notes on anything interesting, useful, confusing, or ridiculous.

1.1.2. Share: Each group will prepare a 5-min presentation (PPT/Word) about your recent experiences using AI for English learning. One member will present in class.

Suggested topics:

- Successful/Failed AI experiences and tips
- Effective prompts for language learning (e.g., CET-4 preparation)
- Appreciation of AI-generated sentences (advanced vocabulary, complex structures, etc.)
- Learning from AI’s mistakes
- Recommendations for new AI tools
- AI-related problems where you need advice from the teacher/peers
- (Feel free to add new topics!)

*Notes: Please include examples (e.g., screenshots, chat history) for clarity. Content originality and authenticity are required.*

1.1.3. Grow: After each presentation, discuss with your group and the teacher to address any challenges you encounter.

## 1.2 Writing Tasks

### 1.2.1 Exposition (Individual in-Class Writing)

A. Instructional focuses:

a. Genre structure: Introduction (with thesis statement) + Body Paragraphs + Conclusion

b. Essay developmental strategies: definition, process, classification, comparison and contrast, examples, cause-effect.

c. GenAI training workshops and prompt template examples:

a). For brainstorming essay topics: “Act as an English writing tutor. I’m an intermediate student (CEFR B1 level) needing expository essay topics about technology’s societal impacts. Propose 5 balanced ideas (both pros/cons) avoiding technical jargon and extreme positions. Simplify complex debates (e.g., privacy vs. convenience) for English learners. Generate 8 draft topics first, then pick the top 5 with clearest conflicts. Format as numbered items with: Topic (e.g., ‘Smartphones in Schools’); one-sentence scope (e.g., ‘Examine focus vs. distraction’); key terms (e.g., ‘productivity, addiction, policy’).”

b). For gathering ideas and seeking out appropriate sources: “Act as an assistant to help me gather expository writing ideas and reliable sources for the topic: Technology and Its Impact on Society. I’m an intermediate EFL student (CEFR B1 level) aiming to analyze both positive and negative effects. Suggest 3-5 focused subtopics (e.g., social media, AI, automation) with brief explanations and 1-2 credible sources per idea (e.g., academic articles, reputable news outlets, or reports). Avoid overly complex terminology. Format ideas in bullet points, include source links, and keep explanations under 50 words each.”

c). For post-writing editing (seeking feedback/overall score): “Act as an expert EFL writing evaluator for my intermediate-level (CEFR B1) essay on [topic]. Provide feedback on Task Achievement/Content aligned with the rubric: [Task Achievement/Content (33%)：Address appropriately and explore the writing prompt in depth; answer the question/s directly by presenting a clear and well-developed thesis statement; present relevant ideas, fully extend and precisely support them with evidence]. Here is my essay: [essay]. Please provide a table summarizing scores, key issues, and quotes from my essay.”

B. Task directions: Based on your chosen topic and your hand-written outlines, please write an exposition (more than 250 words) in 45 minutes.

Topics:

-The Role of Artificial Intelligence (AI) in Healthcare

-The Role of Traditional Chinese Medicine (TCM) in Modern Hospitals

-The Role of Social Media in Students’ Mental Health

-The Role of Study Apps in Academic Productivity

-What Is So Special About Medical Education

-What Is So Special About Being a Doctor (or Nurse)

C: Grading criteria:

Task Achievement/Content (33%): Address appropriately and explore the writing prompt in depth; answer the question/s directly by presenting a clear and well-developed thesis statement; present relevant ideas, fully extend and precisely support them with evidence.

Coherence & Cohesion (33%): Information and ideas are logically organized, and there is a clear progression throughout the response; a range of cohesive devices including reference and substitution are used flexibly; paragraphing is used sufficiently and appropriately.

Lexical Resource (17%): A wide range of vocabulary is fluently and flexibly used to convey precise meanings; there is skillful use of idiomatic items with accuracy in word choice and collocation; spelling and word formation are correct.

Grammatical Range & Accuracy (17%): A variety of complex structures is used with flexibility and accuracy; grammar and punctuation are well-managed.

### 1.2.2 Feature Article (Group out-of-Class Writing)

A. Instructional focuses:

a. Genre structure: Headline (title, subtitle) + Main body (hook, focus, details) + Conclusion

b. Workshops on survey and interview techniques

c. GenAI prompt template example:

a). For feature article grading and feedback: “Please act as an expert writing instructor to evaluate my feature article using the attached grading criteria. Provide dimension scores, overall score and priority suggestions.

Please choose the output language: [English/Chinese].

Here is my feature article: Title […]; Main text […].”

B. Task directions: Working with your group, in three weeks, write a feature article more than 900 words based on interviews and/or surveys to investigate an issue (related to current events, campus news, or hot topics, etc.) or a group of people (it can be people with the same occupation/interest/dream/characteristics/background/difficulty/problem).

C: Grading criteria:

Content and Insight (30%): Demonstrates exceptional originality, depth of thought, and relevance; explores the topic meaningfully and provides compelling insights or conclusions.

Structure and Organization (30%): Logically organized with an engaging hook paragraph introducing the background, a clear focus paragraph illustrating the topic, well-developed body paragraphs with details, and a thoughtful conclusion; transitions between ideas are seamless.

Data Collection and Analysis (20%): Demonstrates exceptional effort in collecting and analyzing data (e.g., surveys, interviews); data is accurately and thoroughly integrated into the article, supporting its arguments.

Language and AI Use (10%): Language is polished, precise, and clear, showing strong writing skills; any AI usage is responsible (e.g., brainstorming, proofreading) and supports the student’s unique voice and ideas.

Critical Thinking and Engagement (10%): Exhibits deep engagement with the topic, showing advanced analytical skills and the ability to synthesize data and ideas into meaningful and original perspectives.

### 1.2.3 Argumentation (Individual in-Class Writing)

A. Instructional focuses:

a. Genre structure: Introduction (with thesis statement) + Body paragraphs (with at least one counterargument and refutation) + Conclusion

b. Refutation techniques: refute the affirmative by highlighting negative outcomes from their reasons; refute the negative by suggesting helpful solutions to their concerns.

c. Language aids:

Counterargument starters: The opposing view is that…; one might object here that…; it might seem that…; it might be partially true that…; some critics/opponents/people (may) argue/claim/contend/think/say that…; it is argued that…

Transitions for refutation: however; nevertheless; in contrast; although; conversely

d. GenAI prompt template example:

a). Argumentative essay grading and overall feedback: “Please act as an expert writing instructor, evaluate my argumentative essay using the attached grading criteria. Provide:

1. Dimension Scores (with direct text examples) and overall score

Task Achievement/Content (25-30/18-24/0-17)

Organization, Coherence & Cohesion (25-30/18-24/0-17)

Lexical Resource (17-20/12-16/0-11)

Grammatical Range & Accuracy (17-20/12-16/0-11)

Overall score out of 100

2. Compliment and Improvement

1)Encouraging compliments on the good parts

2)Improvement suggestions:

Urgent Fixes (2 actionable steps)

Enhancements (1 depth-improvement tip)

Please choose the output language: [English/Chinese].

Here is my argumentative essay: Title […]; Main text […].”

B. Task directions: Based on your chosen topic and your hand-written outlines, please write an argumentation with at least one counterargument (more than 250 words) in one hour.

Topics:

-Does algorithmic recommendation bring more benefits or harm?

-Is watching short video movie explanations a valid alternative to watching movies?

-Is the “Influencer Economy” a viable career path for young people?

-Does social media enhance or harm romantic relationships?

-Do romantic relationships in college hinder or enhance personal development?

-Does English-Medium Instruction (EMI) education bring more benefits or burdens to students?

-Should sex education be included in primary school?

-… (you could create your own argumentative topic)

C: Grading criteria:

Task Achievement/Content (30%): Address appropriately and explore the prompt in depth; Introduction paragraph presents relevant background information and includes a clear thesis statement (author’s opinion and reasons); Body paragraphs present relevant explanations and evidence that logically support the reasons, and include at least one counterargument and effective refutation in the body paragraphs; Conclusion paragraph rephrases the thesis statement, summarizes the main ideas, and concludes with a meaningful final thought/implication.

Coherence & Cohesion (30%): Information and ideas are logically organized, and there is a clear progression throughout the response; a range of cohesive devices including reference and substitution are used flexibly; paragraphing is used sufficiently and appropriately.

Lexical Resource (20%): A wide range of vocabulary is fluently and flexibly used to convey precise meanings; there is skillful use of idiomatic items with accuracy in word choice and collocation; spelling and word formation are correct.

Grammatical Range & Accuracy (20%): A variety of complex structures is used with flexibility and accuracy; grammar and punctuation are well-managed.

## 1.3 Reflective Reports

### 1.3.1 Exposition (Individual Report)

Directions: Please share your honest thoughts on using AI (GAI) for writing – no right or wrong answers! Feel free to express yourself and share critiques or praise in English (encouraged) or Chinese. No word limits. We value real examples and your insights!

(1) Which AI tool/tools did you use for this project? How did you feel about it/them?

(2) What have you learned from AI during this project? Please provide examples. (E.g., idea/content/logic/language/critical thinking/cultural differences, etc.)

(3) What challenges have you faced during this project? Please provide examples.

(4) Compare your first and final drafts: What changes came from AI? What changes came from YOU? (Example: "AI fixed my grammar, but I rewrote the conclusion myself because…")

(5) Share one AI feedback you strongly agreed with and used in your writing. Why did you make this choice?

(6) Share one AI feedback you ignored. Why did you make this choice?

(7) What will you do differently next time when using AI for writing?

(8) Finish this sentence: "AI is like a ____________ for my writing because_______________________."

(9) How do you feel about using AI in this course? (Excited? Confused? Other feelings?)

(10) Any other thoughts/feelings/suggestions?

### 1.3.2 Feature article (Group Report)

Directions: Group reflection: Any group members are welcome to share your honest thoughts– no right or wrong answers! Feel free to express yourself and share critiques or praise in English (encouraged) or Chinese. No word limits. We value real examples and your insights!

(1) Which AI tool(s) supported your feature writing project? In what ways (e.g., brainstorming, researching, editing) did they help? How did you feel about it/them?

(2) Share at least one AI answer that you strongly agreed with and used in your writing. Why did you make this choice?

(3) Share at least one AI answer that you ignored/modified. Why did you make this choice?

(4) How do you feel about using AI in the feature writing project? (Excited? Confused? Worried? Other feelings?)

(5) Any other thoughts/feelings/suggestions?

### 1.3.3 Argumentation (Individual Report)

Directions for reflective summary: Please share your honest thoughts on completing this writing (in English, ＞100 words). We value real examples and your insights (see the sample on the PPT slides)!

Guided questions:

(1) Which AI tool(s) did you use in the whole writing process (before and after writing)? In what ways (e.g., brainstorming, researching, editing) did they help? How did you feel about it/them?

(2) How do you feel about using AI + teacher + peer feedback in this revision? Are they helpful/effective? Are any of them NOT helping?

(3) In this revision experience, what have you learned (e.g., any skills/new words/grammar rules, etc.)? How would you apply what you’ve learned in the coming exams (e.g., CET 4, final exam)?

(4) Any other thoughts/feelings/suggestions?

# 2 Supplementary Table

**Supplementary Table 1.** Coding scheme for the individual and environmental factors contributing to student engagement evolution.

| **Theme** | **Sub-theme** | **Code** | **Example from raw data** |
| --- | --- | --- | --- |
| Individual factors—Lived experiences | Mindsets | a. Growth mindset* | Leo: *GenAI has boosted my enthusiasm for learning English this semester. As I kept getting corrections from GenAI, I became more aware of my own weaknesses. And once I recognized those weaknesses, I felt motivated to address them. I’d say it created a pretty positive cycle.*  (Informal chat 1) |
|  |  | b. Fixed mindset* | Sarah: *The first time I wrote an exposition, I thought it was really bad, and later the score turned out to be quite low. I felt really disappointed. So this time when I had to write again, I just wanted to avoid it.*  (Interview 3.3) |
|  | Beliefs | a. Belief in authorship | Leo: *Simply using GenAI to generate essays isn't really acceptable. You need to ensure your own originality, since it's just a tool to assist.*  (Interview 1.1) |
|  |  | b. Belief in GAI’s usefulness | Leo: *It is quite professional and practical, especially in the field of English.*  (Reflective report 1.1) |
|  |  | c. Belief in self-writing competence | Grace: *Reading GenAI-generated text makes me feel really insecure. I’ve been learning English for so long, and it’s not like I don’t have access to resources, but I still can’t reach that level…it makes me feel like my English is pretty poor.* (Interview 2.1) |
|  |  | d. Belief in GAI’s infallibility | Grace: *I trusted GenAI way too much with grammar, only to find out it could actually make grammatical errors. Unbelievable!…I don’t trust it anymore.*  (Interview 2.3) |
|  |  | e. Belief in GAI’s ease of use | Sarah: *It feels like GenAI can be too much trouble. You have to prompt it in detail every time.*  (Interview 3.3) |
|  | Personal expectancies/values | a. Expectancies for success in writing tasks* | Sarah: *The first time I wrote an exposition, I thought it was really bad, and later the score turned out to be quite low. I felt really disappointed. So this time when I had to write again, I just wanted to avoid it.*  (Interview 3.3) |
|  |  | b. Intrinsic value* | Leo: *I do have a natural interest in language…for example, I enjoy watching American TV series, where I can really appreciate the beauty of the language.*  (Interview 1.1) |
|  |  | c. Utility value* | Grace: *I only focus on practicing English when I’m preparing for exams like CET-4 or CET-6. If there’s no exam coming up, it doesn’t seem necessary.*  (Interview 2.2) |
| Individual factors—Developmental competencies | English proficiency | a. Advanced | Determined using students' class performance, essay scores, and final exam scores from the previous semester |
|  |  | b. Intermediate |  |
|  |  | c. Beginner |  |
|  | Cognitive skills | a. Cognitive skills* | Leo: *(I ignored GenAI’s feedback when) it changed a core word I used. Because it would have lost the connection with the statement if I changed it.*  (Reflective report 1.1) |
|  |  | b. Metacognitive skills* | Leo: *Comprehensive feedback can give me different insights into my passage, forcing me to consider what I wrote from different perspectives. However, feedback from different individuals might contradict with others. We should deliberate on it based on our personal judgement and preference.*  (Reflective report 1.3) |
|  | Emotion regulation/control | a. Adaptive emotion regulation strategies (e.g., cognitive reappraisal, acceptance, problem-solving)* | Leo: *Anxiety isn’t necessarily a bad thing. It can actually help improve your writing.*  (Interview 1.3)  *As I kept getting corrections from GenAI, I became more aware of my own weaknesses. And once I recognized those weaknesses, I felt motivated to address them. I’d say it created a pretty positive cycle.*  (Informal chat 1) |
|  |  | b. Maladaptive emotion regulation strategies (e.g., avoidance, suppression, rumination)* | Sarah: *The first time I wrote an exposition, I thought it was really bad, and later the score turned out to be quite low. I felt really disappointed. So this time when I had to write again, I just wanted to avoid it…Leaving the classroom to hide in the bathroom is also something I did back in middle school and high school.*  (Interview 3.3) |
|  | GAI literacy | a. Developing GAI literacy* | Leo: *When GenAI gives suggestions, especially subjective ones, you need to look at them critically. When it suggests subjective structural changes, it's better to trust your own judgment.*  (Interview 2.2) |
|  |  | b. Undeveloped GAI literacy* | Sarah: *I use BIMT for translation because GenAI might translate my Chinese into something weird…it’s troublesome to write prompts…I didn’t really find the GenAI workshops useful because I had difficulty understanding them.*  (Interview 3.3) |
| Environmental factors—Specific learning tasks/activities | The type of writing task | a. Individual/Collaborative writing | Course syllabus |
|  |  | b. In-class/Out-of-class writing |  |
|  |  | c. Writing genre (exposition/feature article/argumentation) |  |
|  | Evaluation criteria of GAI-assisted writing | a. Essay grading rubrics |  |
|  |  | b. Assignment requirements (first/final drafts; reflective reports; GAI-student chatlogs) |  |
| Environmental factors—The classroom setting | Instructional focuses | a. Essay development |  |
|  |  | b. GAI workshops |  |
|  |  | c. GAI discussion sessions |  |
|  | Social relationships (student-teacher/peer) | a. Student-teacher social relationships | Sarah: *Because the teacher is an educator, I need feedback that is human-level and appropriate for my learning environment.*  (Informal chat 3) |
|  |  | b. Student-peer social relationships | Sarah: *I* *don’t see why classmates need to read my essay. I’d rather not have others see it.*  (Informal chat 3) |
| Environmental factors—The school environment | School norms (learning assessment) | a. English as a compulsory subject for non-English-major undergraduates | Course syllabus and school policy documents |
|  | School policy (GAI use) | a. Development of an institutional GAI platform and a GAI tool (24H Intelligent Assistant) |  |
|  |  | b. Facilitation in formulating course-specific GAI use policies |  |

*Note: The key concepts outlined above are informed by the following body of literature: Eccles and Wigfield (2002), Koltovskaia et al. (2024), Li (2025), Li et al. (2022), Martínez-Priego et al. (2024), and Wang and Xue (2022). See the reference list for further details.
